# Update of the Planktonic Diatom Genus *Pseudo-nitzschia* in Aotearoa New Zealand Coastal Waters: Genetic Diversity and Toxin Production

**DOI:** 10.3390/toxins13090637

**Published:** 2021-09-10

**Authors:** Tomohiro Nishimura, J. Sam Murray, Michael J. Boundy, Muharrem Balci, Holly A. Bowers, Kirsty F. Smith, D. Tim Harwood, Lesley L. Rhodes

**Affiliations:** 1 Cawthron Institute, Nelson 7010, New Zealand; sam.murray@cawthron.org.nz (J.S.M.); michael.boundy@cawthron.org.nz (M.J.B.); kirsty.smith@cawthron.org.nz (K.F.S.); tim.harwood@cawthron.org.nz (D.T.H.); 2 Biology Department, Faculty of Science, Istanbul University, Istanbul 34134, Turkey; muharrem.balci@istanbul.edu.tr; 3 Moss Landing Marine Laboratories, Moss Landing, CA 95039, USA; hbowers@mlml.calstate.edu; 4 School of Biological Sciences, University of Auckland, Auckland 1142, New Zealand

**Keywords:** amnesic shellfish poisoning, domoic acid, geographical distribution, liquid chromatography-tandem mass spectrometry, molecular phylogenetic analysis, Pacific Ocean, phytoplankton

## Abstract

Domoic acid (DA) is produced by almost half of the species belonging to the diatom genus *Pseudo-nitzschia* and causes amnesic shellfish poisoning (ASP). It is, therefore, important to investigate the diversity and toxin production of *Pseudo-nitzschia* species for ASP risk assessments. Between 2018 and 2020, seawater samples were collected from various sites around Aotearoa New Zealand, and 130 clonal isolates of *Pseudo-nitzschia* were established. Molecular phylogenetic analysis of partial large subunit ribosomal DNA and/or internal transcribed spacer regions revealed that the isolates were divided into 14 species (*Pseudo-nitzschia americana*, *Pseudo-nitzschia arenysensis*, *Pseudo-nitzschia australis*, *Pseudo-nitzschia calliantha*, *Pseudo-nitzschia cuspidata*, *Pseudo-nitzschia delicatissima*, *Pseudo-nitzschia fraudulenta*, *Pseudo-nitzschia galaxiae*, *Pseudo-nitzschia hasleana*, *Pseudo-nitzschia multiseries*, *Pseudo-nitzschia multistriata*, *Pseudo-nitzschia plurisecta*, *Pseudo-nitzschia pungens*, and *Pseudo-nitzschia* cf. *subpacifica*). The *P. delicatissima* and *P. hasleana* strains were further divided into two clades/subclades (I and II). Liquid chromatography-tandem mass spectrometry was used to assess the production of DA and DA isomers by 73 representative strains. The analyses revealed that two (*P. australis* and *P. multiseries*) of the 14 species produced DA as a primary analogue, along with several DA isomers. This study is the first geographical distribution record of *P. arenysensis*, *P.* *cuspidata*, *P. galaxiae*, and *P. hasleana* in New Zealand coastal waters.

## 1. Introduction

The first amnesic shellfish poisoning (ASP) outbreak, caused by consumption of cultivated blue mussels, occurred in Canada in 1987, and several deaths were recorded [1,2,3]. At the same time, *Pseudo-nitzschia multiseries* (formerly reported as *Nitzschia pungens* f. *multiseries*) was identified as the causative species [1,2,3,4]. It produced a biotoxin, domoic acid (DA) [2], which has been monitored in Aotearoa New Zealand shellfish since 1993 [5], and is currently the only DA analogue regulated in New Zealand [6,7]. Additional DA isomers have been reported to date [8], however there is insufficient toxicity information to establish Toxicity Equivalency Factors (TEF) for the isomers [9]. As well as shellfish being tested, seawater samples are analysed to determine the presence and quantity of *Pseudo-nitzschia* cells, which bloom regularly in the coastal waters, to allow for risk assessments for the shellfish industry and regulators [10]. These assessments aid managers in their harvesting decisions and help public health officials determine whether to post warnings for recreational harvesters of potential toxins in seafood.

The occurrence of *Pseudo-nitzschia* blooms in New Zealand was summarised in 2000 [5] and again in 2011 [10]. An overview of harmful algal blooms (HABs) in Australasia, a region comprising Australia, New Zealand, and some neighbouring islands, then reviewed the monitoring data for *Pseudo-nitzschia* and its toxins up to 2018 [11]. Despite intensive monitoring of shellfish since 1993, only one critically high DA concentration has been reported, in scallop digestive glands in 1994, following the collapse of a *P. australis* bloom in Bream Bay, Northland, New Zealand [12]. More recently, a high DA concentration above regulatory level has been reported in Queen scallop flesh collected in deep water off the coast of Otago, New Zealand in December 2020 (Harwood et al. unpublished data). No human illnesses due to DA poisoning have occurred by consumption of shellfish harvested in New Zealand to dateto date, due to the monitoring efforts.

By 2011, there were 35 *Pseudo-nitzschia* species known globally (14 of which were DA producers) and, of those species, 12 were recorded in New Zealand at that time (seven of which were known DA producers). Those species were *P. americana*, *P. australis*, *P. caciantha*, *P. calliantha*, *P. delicatissima* (including a genotype formerly reported as ‘*Pseudo-nitzschia* *turgidula*’), *P. fraudulenta*, *P. multiseries*, *P. multistriata*, *P. plurisecta* (formerly reported as *P. cuspidata* Hobart-5 type), *Pseudo-nitzschia pseudodelicatissima*, *P. pungens*, and *P. subpacifica* [formerly reported as *Pseudo-nitzschia* (cf.) *heimii*] [10]. Over the last decade, 23 additional species have been described globally (12 of those being known DA producers), resulting in 58 species as of 2021 [13], with 26 of those being DA producers.

The current study aims to refresh the data on the diversity of and toxin production by *Pseudo-nitzschia*, by presenting the results following the isolation of more than 100 *Pseudo-nitzschia* uni-cells/chains from the coastal waters of New Zealand’s three main islands. Analysis of molecular phylogenetic data from these isolates enables reporting of newly recorded species of *Pseudo-nitzschia* in New Zealand. This new information, which includes toxin profiles, will allow risk assessments to be refined in New Zealand.

## 2. Results

### 2.1. Molecular Phylogenetic Characteristics

#### 2.1.1. Molecular Phylogeny

Genotype screening using molecular phylogenetic analysis based on the nuclear-encoded ribosomal RNA gene (rDNA), specifically, internal transcribed spacer (ITS) 1–5.8S rDNA–ITS 2 region (ITS region) and large subunit (LSU) rDNA D1–D3 region (LSU rDNA D1–D3), was conducted for a total of 130 isolates of *Pseudo-nitzschia* established from 22 sites in subtropical and temperate coastal waters in New Zealand (Table S1). The screening revealed that the strains could be separated into 14 species: *P. americana* [number of strains (*n*) = 6], *P. arenysensis* (*n* = 7), *P. australis* (*n* = 14), *P. calliantha* (*n* = 3), *P. cuspidata* (*n* = 1), *P. delicatissima* (*n* = 28), *P. fraudulenta* (*n* = 13), *P. galaxiae* (*n* = 2), *P. hasleana* (*n* = 6), *P. multiseries* (*n* = 4), *P. multistriata* (*n* = 13), *P. plurisecta* (*n* = 1), *P. pungens* (*n* = 27), and *P.* cf. *subpacifica* (*n* = 5) (Table S2). Furthermore, the strains of five species (*P. arenysensis*, *P. cuspidata*, *P. delicatissima*, *P. galaxiae*, *P. hasleana*, and *P. pungens*) belonged to one or two clades/subclades as explained below.

**Figure 1:**
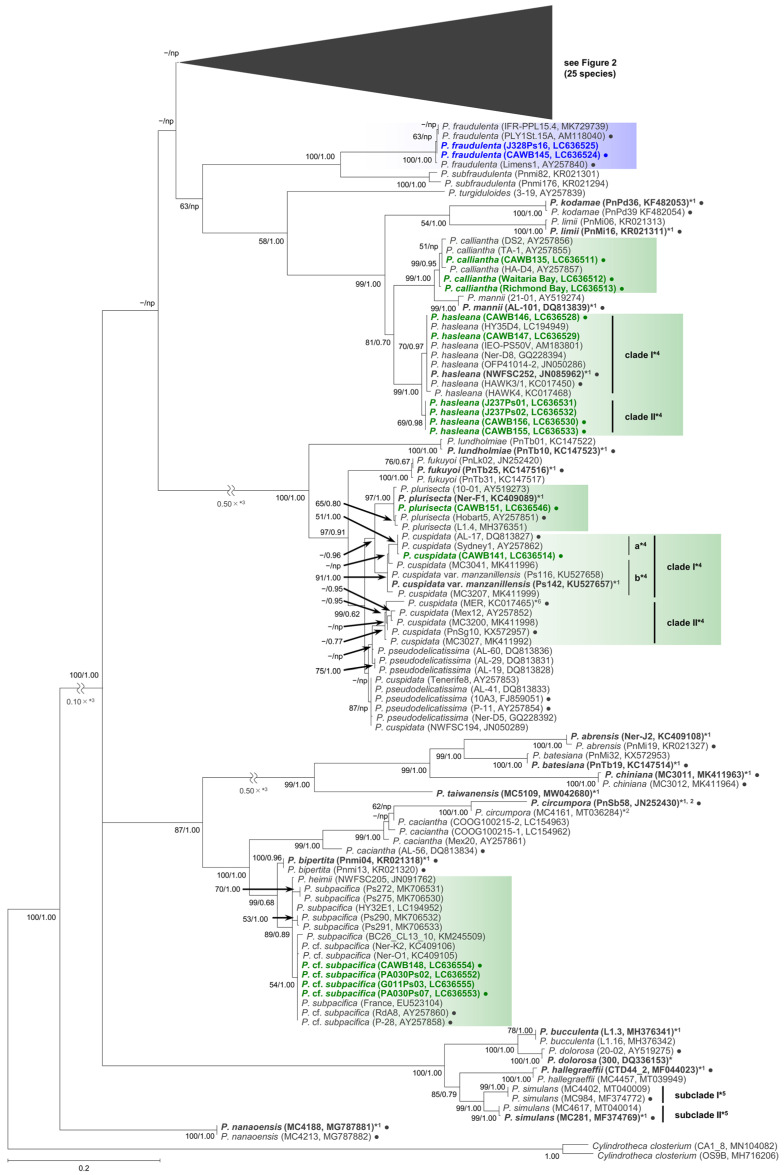
Molecular phylogenetic tree of 52 *Pseudo-nitzschia* species based on the ITS region sequences (253 sequences, 809 positions) using maximum likelihood (ML) analysis. See Figure 2 for details of 25 species. Strains from New Zealand are shown in colour fonts. Blue and green fonts indicate potential ‘low and no ASP risk’ species in New Zealand, respectively. A Black or coloured circle indicates a strain used in the LSU rDNA D1–D3 tree shown in Figure 3. Nodal support represents ML bootstrap value/Bayesian inference (BI) posterior probability. Nodal support under 50 in ML or 0.50 in BI is shown as a minus sign (−). A node that was not present in the BI tree is labelled as np. A scale bar indicates the number of nucleotide substitutions per site. *^1^: A sequence obtained from holotype material is shown in bold font. *^2^: A sequence having only ITS 2 region. *^3^: A reduction ratio of reduced nodal length calculated from original nodal length. *^4^: Clade separation reported by the present study. *^5^: Subclade separation reported by Ajani et al. (2020) [17]. *^6^: *Pseudo-nitzschia cuspidata* strain MER was re-assigned as *P.* cf. *cuspidata* by Ajani et al. (2021) [18].

**Figure 2:**
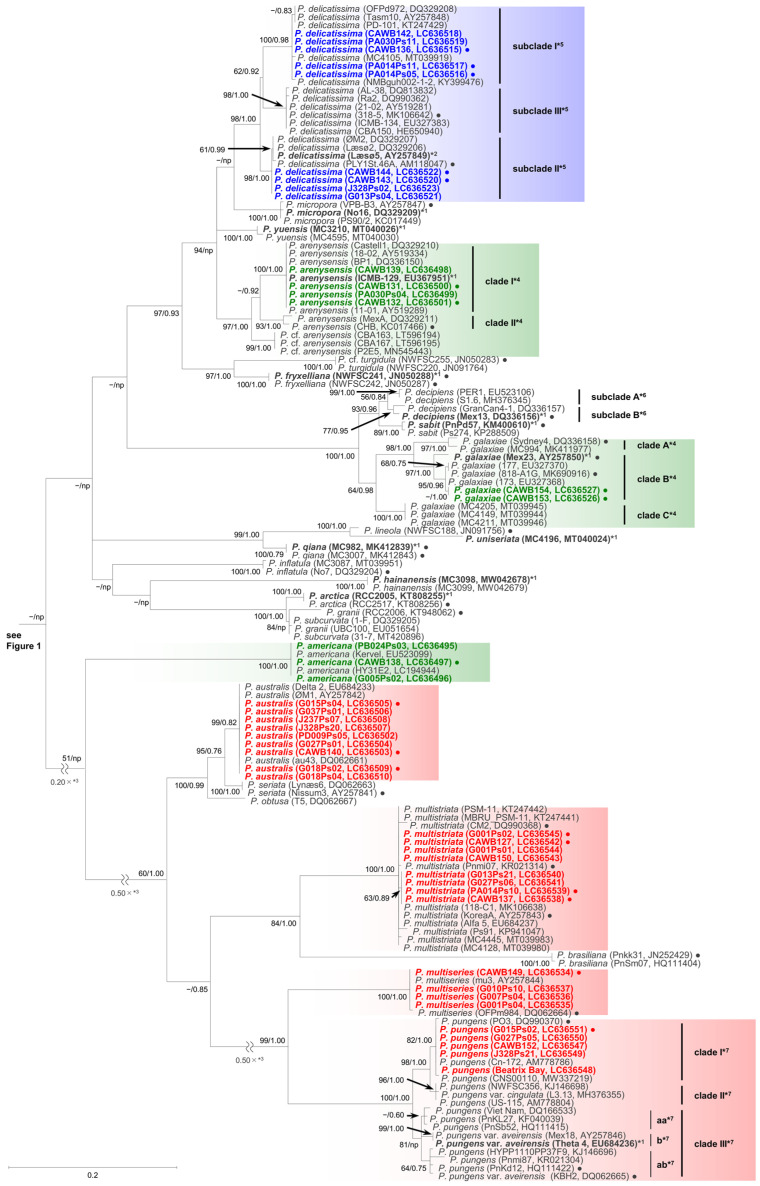
Molecular phylogenetic tree of 25 *Pseudo-nitzschia* species based on the ITS region sequences (144 sequences, 809 positions) using maximum likelihood (ML) analysis. Strains from New Zealand are shown in colour fonts. Red, blue, and green fonts indicate potential ‘high, low, and no ASP risk’ species in New Zealand, respectively. A Black or coloured circle indicates a strain used in the LSU rDNA D1–D3 tree shown in Figure 3. Nodal support represents ML bootstrap value/Bayesian inference (BI) posterior probability. Nodal support under 50 in ML or 0.50 in BI is shown as a minus sign (−). A node that was not present in the BI tree is labelled as np. A scale bar indicates the number of nucleotide substitutions per site. *^1^: A sequence obtained from holotype material is shown in bold font. *^2^: A sequence obtained from epitype material is shown in bold font. *^3^: A reduction ratio of reduced nodal length calculated from original nodal length. *^4^: Clade separation reported by the present study. *^5^: Subclade separation reported by Stonik et al. (2018) [14]. *^6^: Subclade separation reported by Gai et al. (2018) [15]. *^7^: Clade separation reported by Kim et al. (2015) [16].

**Figure 3:**
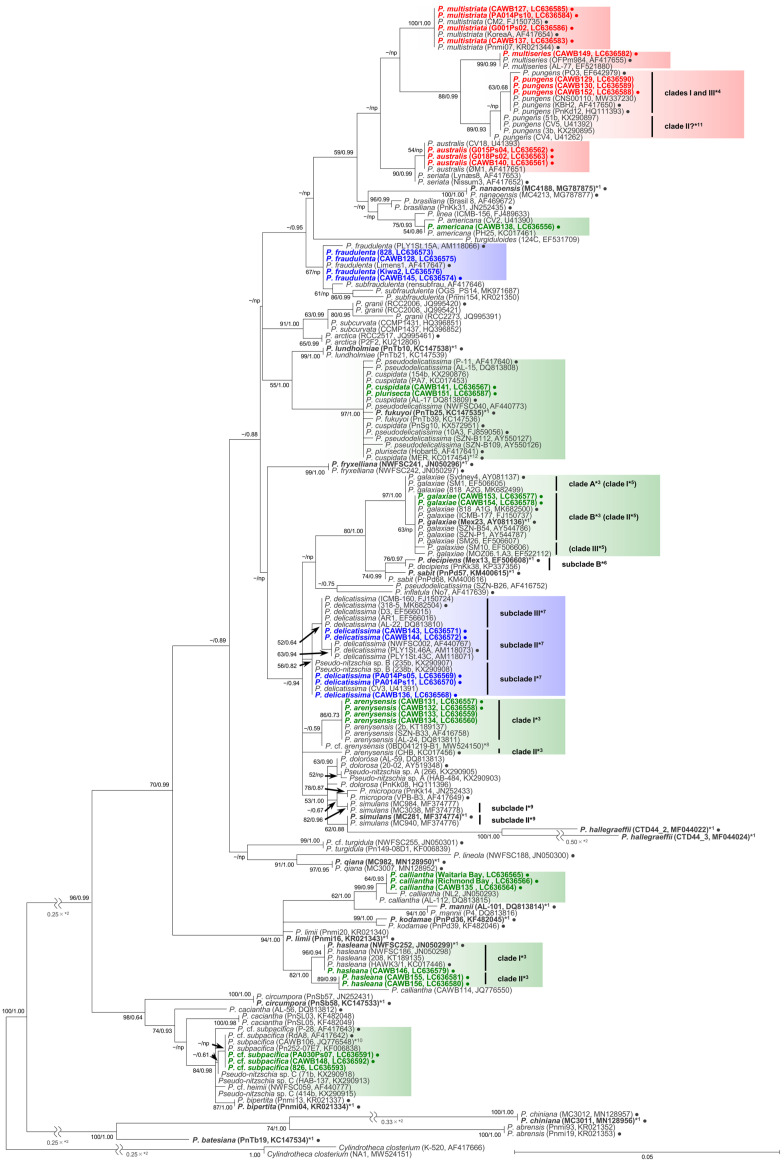
Molecular phylogenetic tree of 47 *Pseudo-nitzschia* species based on the LSU rDNA D1–D3 sequences (179 sequences, 660 positions) using maximum likelihood (ML) analysis. Strains from New Zealand are shown in colour fonts. Red, blue, and green fonts indicate potential ‘high, low, and no ASP risk’ species in New Zealand, respectively. A Black or coloured circle indicates a strain used in the ITS region trees shown in Figure 1 and Figure 2. Nodal support represents ML bootstrap value/Bayesian inference (BI) posterior probability. Nodal support under 50 in ML or 0.50 in BI is shown as a minus sign (−). A node that was not present in the BI tree is labelled as np. A scale bar indicates the number of nucleotide substitutions per site. *^1^: A sequence obtained from holotype material is shown in bold font. *^2^: A reduction ratio of reduced nodal length calculated from original nodal length. *^3^: Clade separation reported by the present study. *^4^: Clade separation reported by Kim et al. (2015) [16]. *^5^: Clade separation reported by McDonald et al. (2007) [19]. *^6^: Subclade separation reported by Gai et al. (2018) [15]. *^7^: Subclade separation reported by Stonik et al. (2018) [14]. *^8^: Strain 0BD041219-B1, originally reported as *P. delicatissima* by Dermastia et al. (unpublished data), is assigned as *P.* cf. *arenysensis* as its sequence was almost identical to those of *P.* cf. *arenysensis* reported recently by Giulietti et al. (2021) [20]. *^9^: Subclade separation reported by Ajani et al. (2020) [17]. *^10^: *Pseudo-nitzschia* cf. *heimii* strain CAWB106 is assigned as *P. subpacifica* based on morphological characters following discussions in Rhodes et al. (2013) [10]. *^11^: *Pseudo-nitzschia pungens* strains CV4, CV5, 3b, and 51b [21,22] were isolated from California, USA where only *P. pungens* clade II was reported to date. These strains were tentatively assigned as putative clade II of *P. pungens* in the present study. *^12^: *Pseudo-nitzschia cuspidata* strain MER was re-assigned as *P.* cf. *cuspidata* by Ajani et al. (2021) [18].

As the sequences of the strains of each species/clade/subclade had high levels of identity, selected representative sequences from each species/clade/subclade were used for the molecular phylogenetic analysis using the maximum likelihood (ML) and Bayesian inference (BI) methods. The number of the representative sequences for the ITS region and LSU rDNA D1–D3 were 61 and 38, respectively (Table S2). The ITS region phylogeny comprising 52 *Pseudo-nitzschia* species is shown in Figure 1 and Figure 2. The LSU rDNA D1–D3 tree comprising 47 *Pseudo-nitzschia* species is shown in Figure 3.

In the phylogenetic trees, eight *Pseudo-nitzschia* species (*P. arenysensis*/*P.* cf. *arenysensis*, *P. cuspidata*, *Pseudo-nitzschia decipiens*, *P. delicatissima*, *P. galaxiae*, *P. hasleana*, *P. pungens*, and *Pseudo-nitzschia simulans*) could be separated into various clades or subclades (Figure 1, Figure 2 and Figure 3). Among these clades/subclades, the New Zealand strains belonged to *P. arenysensis* clade I, *P. cuspidata* clade Ia, *P. delicatissima* subclades I and II, *P. galaxiae* clade B, *P. hasleana* clades I and II, and *P. pungens* clade I (Figure 1, Figure 2 and Figure 3). The clade/subclade separation previously reported for *P. delicatissima* [14], *P. decipiens* [15], *P. pungens* [16], and *P. simulans* [17] were applied in the present study. The clade/subclade separation for *P. arenysensis*, *P. cuspidata*, *P. galaxiae*, and *P. hasleana* were proposed in the present study. Among them, *P. hasleana* clade II was unreported so far and firstly reported by the present study. Although all species/clades/subclades, except for combinations of *Pseudo-nitzschia granii*/*Pseudo-nitzschia subcurvata* and *P.* (cf.) *heimii*/*P.* (cf.) *subpacifica*, were separated in the ITS trees (Figure 1 and Figure 2), the sequences belonging to two combinations (*P. pungens* clades I/III and *P. cuspidata*/*Pseudo-nitzschia fukuyoi*/*P. plurisecta*/*P. pseudodelicatissima*) were clustered together respectively in the LSU rDNA D1–D3 tree (Figure 3).

#### 2.1.2. Sequence Analysis

To reveal genetic divergence, especially for the newly reported *P. hasleana* clade II, the uncorrected *p* distances of the ITS region sequences of selected combinations between *Pseudo-nitzschia* species/clades/subclades were calculated. The selected species were *P. arenysensis*/*P.* cf. *arenysensis*, *P. cuspidata*/*P. pseudodelicatissima*/*P. plurisecta*, *P. decipiens*, *P. delicatissima*, *P. galaxiae*, *P. hasleana*, *P. pungens*, and *P. simulans* (Table S3). The comparisons revealed that the genetic divergence between *P. hasleana* clades I and II (the *p* distance: 0.012 ± 0.001, 0.011–0.015) were the same as that between *P. pungens* clades I and II (0.012 ± 0.001, 0.011–0.014) (Table S3). In contrast, that within *P. cuspidata* clade II (0.012 ± 0.008, 0.003–0.024) was similar to that between *P. cuspidata* clades Ia and Ib (0.013 ± 0.004, 0.010–0.021) (Table S3). Additionally, sequences of four New Zealand strains of *P. delicatissima* subclade II (strains CAWB143, CAWB144, G013Ps04, and J328Ps02) were slightly different (1–2 positions difference in 671 positions) from those of the others (strains ØM2, Læsø2, Læsø5, and PLY1St.46A) belonging to the same subclade (0.002 ± 0.001, 0.002–0.003) (Table S3, Figure 2).

The *p* distances of the LSU rDNA D1–D3 sequences of selected combinations between *Pseudo-nitzschia* species/clades, including *P. hasleana* clade II, were calculated. The selected species/clades were *P. hasleana* clades I and II, ‘*P. calliantha*’, *P. calliantha*, *Pseudo-nitzschia mannii*, *Pseudo-nitzschia limii*, and *Pseudo-nitzschia kodamae* (Table S4). The comparisons revealed that the average *p* distance between *P. hasleana* clades I and II (0.006) were slightly lower than those of other combinations (0.007–0.035) (Table S4). The average *p* distances of ‘*P. calliantha*’ strain CAWB114 towards *P. hasleana* clades I and II and *P. calliantha* were 0.014, 0.008, and 0.027, respectively (Table S4). Additionally, the values of ‘*P. calliantha*’ strain CAWB114 towards *P. hasleana* clades I (0.014) and II (0.008) were higher than or similar to those between clades in the same species [*P. hasleana* clades I and II (0.006)] or between species [*P. hasleana* clade I and *P. limii* (0.007), or *P. hasleana* clade II and *P. limii* (0.008)] (Table S4).

### 2.2. Distribution

**Figure 4:**
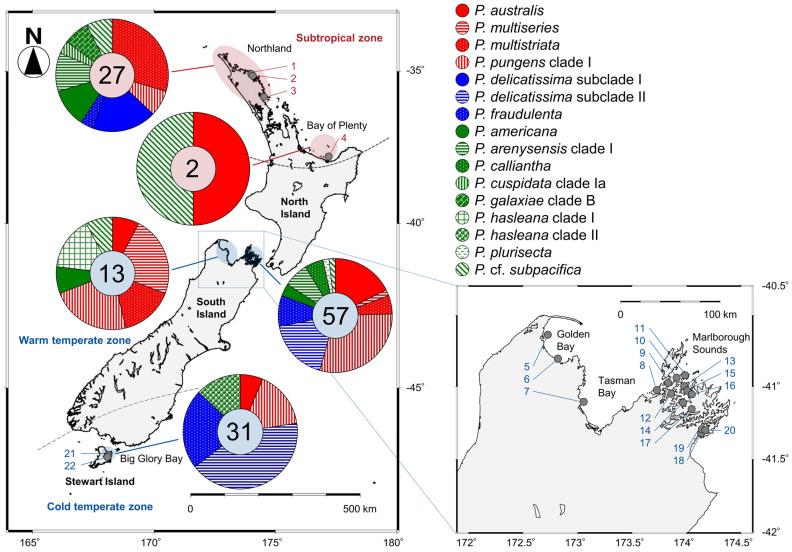
Sampling and distribution map of 14 *Pseudo-nitzschia* species in New Zealand coastal waters assessed between 2018 and 2020. The locations of 22 sampling sites (grey circles) and their site codes are shown. The numbers in each pie chart indicate the total number of clonal strains genetically identified by the molecular phylogenies. Red, blue, and green indicate potential ‘high, low, and no ASP risk’ species in New Zealand, respectively. Sampling details, including sampling site code, are shown in Table S1.

Among the 130 clonal *Pseudo-nitzschia* strains established and genetically identified in the present study, 29 strains originated from four subtropical sites and 101 strains originated from 18 temperate sites (Figure 4 and Table S2). In the subtropical zone, the number of species found from Northland and Bay of Plenty regions were nine and two, respectively. In the temperate zone, those from Golden Bay/Tasman Bay, Marlborough Sounds, and Big Glory Bay (Stewart Island) were seven, eleven, and five, respectively (Figure 4).

The species/clades/subclades composition plotted onto a map suggested that each of them showed a unique distribution pattern in New Zealand coastal waters (Figure 4). *Pseudo-nitzschia americana*, *P. arenysensis* clade I, *P. australis*, *P. fraudulenta*, *P. multistriata*, *P. pungens* clade I, and *P.* cf. *subpacifica* were widespread from subtropical to temperate zones (Figure 4). On the other hand, *P. cuspidata* clade Ia, *P. delicatissima* subclade I, *P. galaxiae* clade B were restricted in the subtropical zone, whereas *P. calliantha*, *P. delicatissima* subclade II, *P. hasleana* clades I and II, *P. multiseries*, and *P. plurisecta* were restricted in the temperate zone (Figure 4). *Pseudo-nitzschia hasleana* clades I and II were distributed in warm temperate and cold temperate zones, respectively (Figure 4).

### 2.3. Toxin Production

#### 2.3.1. Production of Domoic Acid (DA) and Its Isomers

**Figure 5:**
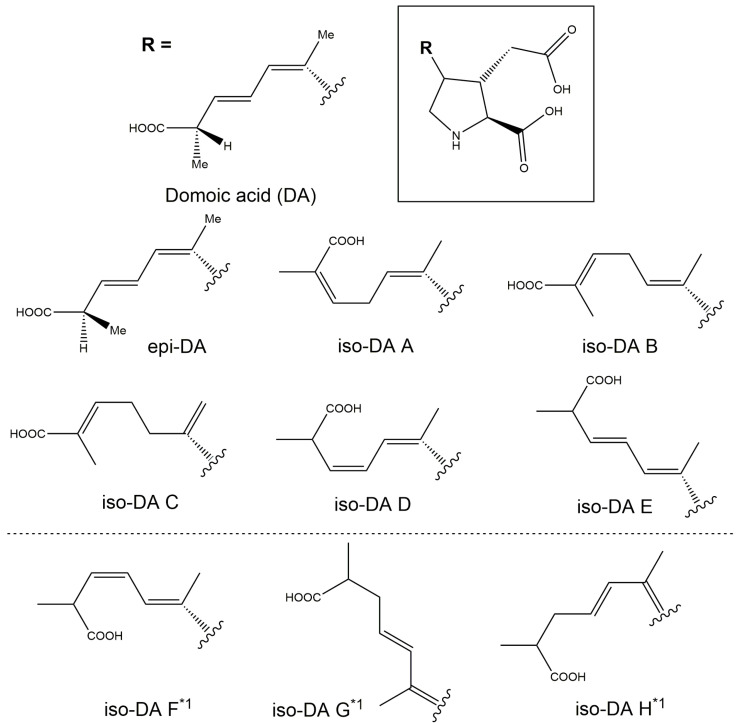
Structures of domoic acid (DA) and DA isomers. Adapted from Quilliam et al. (1995) [23]. *^1^ DA isomers not analysed in the present study.

**Figure 6:**
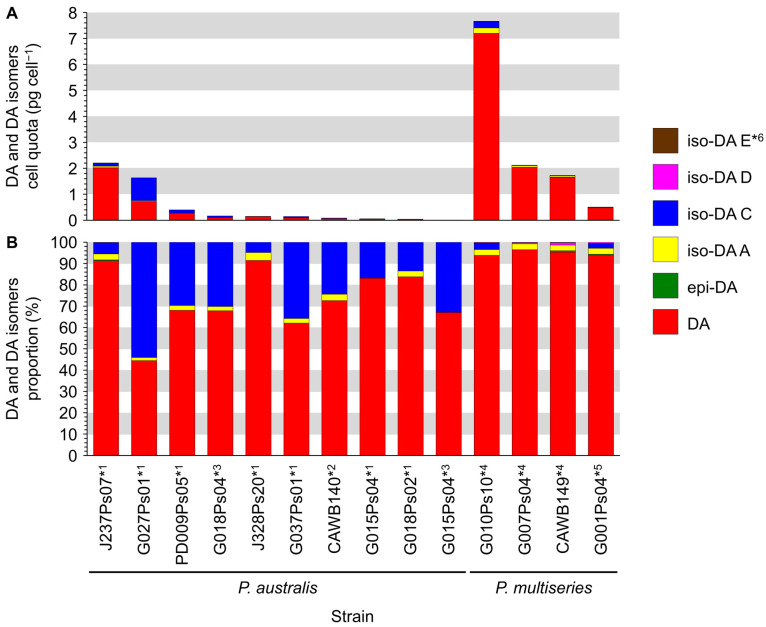
Domoic acid (DA) and DA isomers profiles of *Pseudo-nitzschia australis* and *P. multiseries* strains from New Zealand. (**A**) DA and DA isomers cell quota (pg cell^−1^); (**B**) DA and DA isomers proportion (%). Asterisks: Strain was cultured using f/2−Si medium at *^1^ three, *^2^ four, *^3^ seven, *^4^ nine, or *^5^ ten months after the strain was established. *^6^: Three *P. multiseries* strains (G010Ps10, CAWB149, and G001Ps04) produced very low cell quotas of iso-DA E (0.002–0.005 pg cell^−1^, 0.1–0.2%), resulting in difficulty in seeing these data in Figure 6 (**A**,**B**).

**Figure 7:**
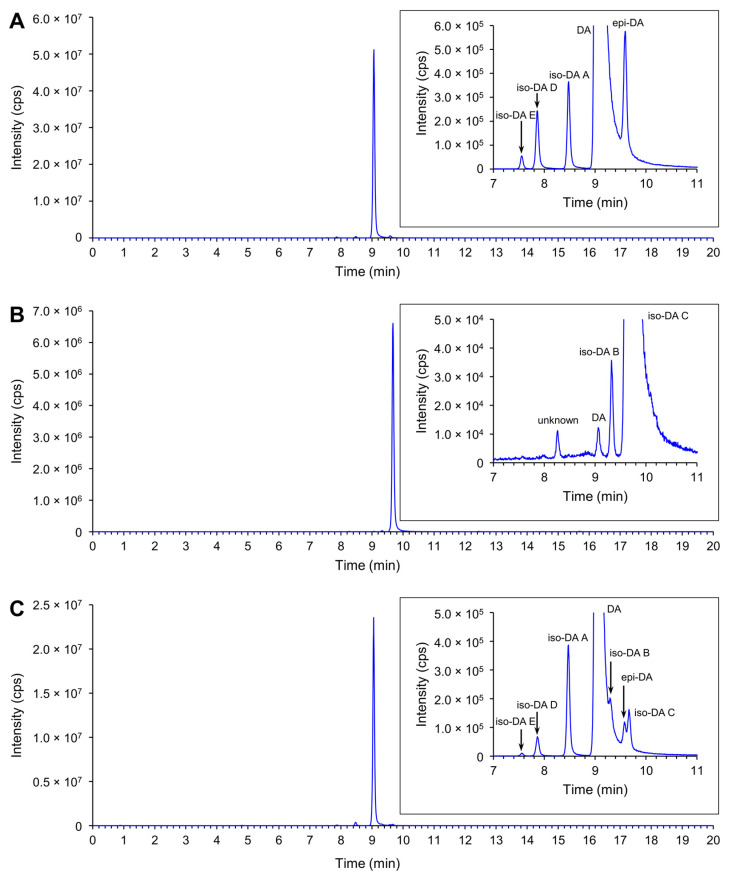
Total ion chromatogram of DA and DA isomers (combined 312 > 266 and 312 > 161) of DA and DA isomers standards and a *Pseudo-nitzschia* extract. (**A**) 1 µL injection of 1/50 dilution of NRCC DA-f DA and DA isomers standard; (**B**) 1 µL injection of iso-DA C standard (950 ng mL^−1^); (**C**) 1 µL injection of an extract of *P. multiseries* strain G001Ps04.

Production of DA and its isomers [c5′-epidomoic acid (epi-DA), isodomoic acids (iso-DAs) A, B, C, D, and E; Figure 5] was assessed in 73 representative strains, from 14 *Pseudo-nitzschia* species genetically identified above (Section 2.1), using liquid chromatography-tandem mass spectrometry (LC-MS/MS). Strain details, including cell quotas of DA and the monitored isomers, expressed as pg cell^−1^, are shown in Table S5. Toxin cell quota detected between the limit of detection (LoD) (0.00005–0.0005 pg cell^−1^) and lower limit of quantitation (LLoQ) (0.0005–0.003 pg cell^−1^) was expressed as ‘trace level’ (Table S5). All the assessed strains of *P. australis* [number of strains (*n*) = 9] and *P. multiseries* (*n* = 4) produced DA with ranges of 0.004–2.02 pg cell^−1^ and 0.47–7.20 pg cell^−1^, respectively (Figure 6A and Table S5). The DA cell quotas of *P. multiseries* strains were higher than those of *P. australis* strains (Mann–Whitney U test, *p* < 0.05). By contrast, the productivity of DA isomers of the strains was different between *P. australis* and *P. multiseries*. All *P. australis* strains produced low quantities of iso-DA A (trace level–0.060 pg cell^−1^), iso-DA B (trace level), and iso-DA C (0.002–0.884 pg cell^−1^), while some strains produced low quantities of epi-DA (trace–0.010 pg cell^−1^), iso-DA D (trace level–0.003 pg cell^−1^) and/or iso-DA E (trace level) (Figure 6A and Table S5). All *P. multiseries* strains produced low quantities of iso-DA A (0.014–0.206 pg cell^−1^), iso-DA D (0.003–0.020 pg cell^−1^), and iso-DA E (trace level–0.005 pg cell^−1^), while some strains produced low quantities of epi-DA (0.002–0.012 pg cell^−1^), iso-DA B (trace level) and/or iso-DA C (0.012–0.239 pg cell^−1^) (Figure 6A and Table S5). Multiple reaction monitoring (MRM) chromatograms of DA and DA isomers standards and an extract of *P. multiseries* strain G001Ps04 are shown in Figure 7. Two out of the eight strains of *P. multistriata* assessed produced trace levels of DA and iso-DA A, and one strain of *P. cuspidata* clade Ia produced a trace level of DA (Table S5). None of the other 57 strains assessed showed detectable concentrations of the DA and DA isomers examined.

#### 2.3.2. Relative Proportion of DA and Its Isomers

The relative proportion of DA and its isomers produced by the *P. australis* and *P. multiseries* strains assessed were compared (Figure 6B). Analogues detected with trace levels were ignored in the calculation. The comparison revealed that the primary analogue produced by the strains of both species was DA, whereas the proportions of DA isomers of the strains differed between *P. australis* and *P. multiseries*. The primary analogue produced by *P. australis* strains was DA (44.4–91.5% of total DA and its isomers quantified). The second primary analogue of the strains was iso-DA C (4.8–54.1%), and the other analogues were minor contributors (epi-DA, 0.4%; iso-DA A, 1.5–3.7%; and iso-DA D, 0.1%) (Figure 6B and Table S5). The primary analogue produced by *P. multiseries* strains was DA (93.9–96.5%), followed by iso-DA A (2.6–2.8%) and iso-DA C (2.3–3.1%), with the other analogues being minor contributors (epi-DA, 0.3–0.7%; iso-DA D, 0.3–1.2%; and iso-DA E, 0.1–0.2%) (Figure 6B and Table S5). The DA proportions of *P. multiseries* strains were higher than those of *P. australis* strains (Mann–Whitney U test, *p* < 0.01).

### 2.4. Potential ASP Risk of Each Pseudo-nitzschia Species in New Zealand Coastal Waters

#### 2.4.1. Classification of Potential ASP Risk for Each Species

The potential ASP risk for the 14 *Pseudo-nitzschia* species morphologically and/or genetically identified and two species morphologically identified previously was classified based on toxin production. As a result, four species (*P. australis*, *P. multiseries*, *P. multistriata*, and *P. pungens*) were classified as potential ‘high ASP risk’ species and three species (*P. delicatissima*, *P. fraudulenta*, and ‘*P. pseudodelicatissima*’) as potential ‘low ASP risk’ species. The other nine species [*P. americana*, *P. arenysensis*, ‘*P. caciantha’*, *P. calliantha*, *P. cuspidata*, *P. hasleana*, *P. galaxiae*, *P. plurisecta*, and *P.* (cf.) *subpacifica*] were classified as potential ‘no ASP risk’ species (Table 1).

#### 2.4.2. Distribution of Each Species

**Figure 8:**
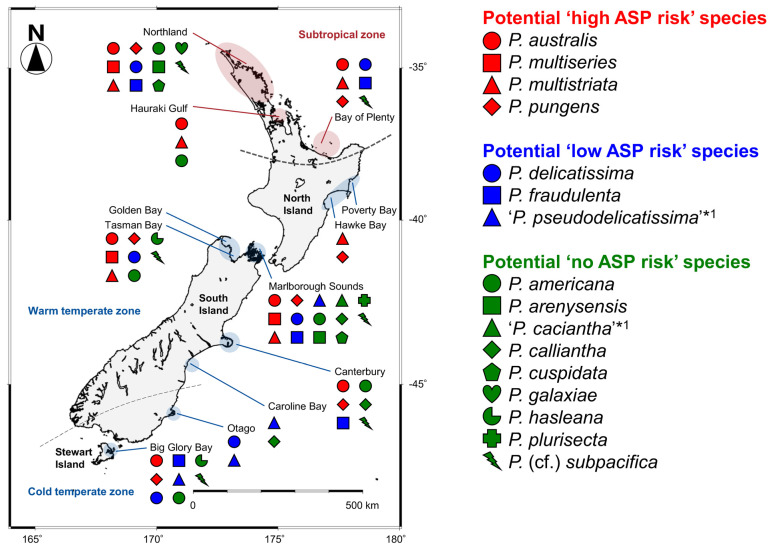
Distribution map of 16 *Pseudo-nitzschia* species in New Zealand coastal waters between 1993 and 2020. Red, blue, and green indicate potential ‘high, low, and no ASP risk’ species in New Zealand, respectively. *^1^: Species identification should be treated as tentative, as discussed in Section 3.1.

The distribution pattern of the 16 *Pseudo-nitzschia* species in New Zealand were summarised and mapped with their potential ASP risk information. The four potential ‘high ASP risk’ species (*P. australis*, *P. multiseries*, *P. multistriata*, and *P. pungens*) were distributed from the subtropical to temperate zones, with no records of *P. multiseries* and *P. multistriata* from the cold temperate zone (Figure 8 and Table 1). Regarding the three potential ‘low ASP risk’ species, *P. delicatissima* and *P. fraudulenta* were distributed from the subtropical to temperate zones, although ‘*P. pseudodelicatissima*’ was restricted in the temperate zone (Figure 8 and Table 1). In terms of the nine potential ‘no ASP risk’ species, four species [*P. americana*, *P. arenysensis*, *P. plurisecta*, and *P.* (cf.) *subpacifica*] were distributed from the subtropical to temperate zones, with no records of *P. arenysensis* and *P. plurisecta* from the cold temperate zone. On the other hand, *P. galaxiae* was restricted to the subtropical zone, while the other four species (‘*P. caciantha*’, *P. calliantha*, *P. cuspidata*, and *P. hasleana*) were restricted to the temperate zone. Among the latter four species, *P. hasleana* was the only species recorded from the cold temperate zone (Figure 8 and Table 1). In detail, *P. hasleana* clade I was recorded from the warm temperate zone, whereas *P. hasleana* clade II was recorded from the cold temperate zone (Figure 4).

## 3. Discussion

### 3.1. Diversity of Pseudo-nitzschia Species in New Zealand

Among the current 58 *Pseudo-nitzschia* species in the literature, 57 species are accepted taxonomically. However, the remaining species, *Pseudo-nitzschia* *sinica*, was claimed to be an invalid name because its type was not indicated so far [24]. Historically, morphological characterisations were conducted, and all species were described with their specific morphological features. More recently, molecular phylogenetic characterisations have been added as an additional tool to classify *Pseudo-nitzschia* species. Ribosomal DNA [small subunit (SSU) rDNA; LSU rDNA; and ITS region], the mitochondrial encoded cytochrome *c* oxidase subunit 1 (*cox1*) gene, the chloroplast encoded Ribulose-1,5-bisphosphate carboxylase-oxygenase (RuBisCO) large subunit (*rbcL*) gene, and the RuBisCO small subunit (*rbcS*) gene have been used so far [25,26]. Among these gene regions, the rDNA regions, especially LSU rDNA D1–D3 and ITS region, are frequently used for molecular characterisation. The latter region, having a higher resolution than the former [27], can distinguish almost all *Pseudo-nitzschia* species whose sequences have been reported so far. Furthermore, sequences of several recently described species were reported with only the ITS region and not together with the LSU rDNA D1–D3 [13,15,28]. Additionally, among the 58 species, sequences of five species (*Pseudo-nitzschia* *antarctica*, *Pseudo-nitzschia* *prolongatoides*, *Pseudo-nitzschia* *pungiformis*, *Pseudo-nitzschia* *roundii*, and *P. sinica*) have not yet been determined. To date, sequences of 52 and 47 *Pseudo-nitzschia* species have been deposited in the International Nucleotide Sequence Database Collaboration [the DNA Data Bank of Japan (DDBJ)/the European Molecular Biology Laboratory Nucleotide Sequence Database (EMBL)/GenBank] for the ITS region and the LSU rDNA D1–D3, respectively. To complete the sequence data set of both regions, sequences of the five morphologically described species above, as well as the ITS region of *Pseudo-nitzschia* *linea* and the LSU rDNA D1–D3 of six species (*Pseudo-nitzschia* *bucculenta*, *Pseudo-nitzschia* *hainanensis*, *Pseudo-nitzschia* *obtusa*, *Pseudo-nitzschia* *taiwanensis*, *Pseudo-nitzschia* *uniseriata*, and *Pseudo-nitzschia* *yuensis*), still need to be determined.

The ITS region is considered a suitable marker for resolving *Pseudo-nitzschia* species classification. We used the ITS region for genotype screening of the clonal isolates established from New Zealand coastal waters. Previously, sequences from New Zealand isolates were reported for the ITS region of *P. pungens* clade I [29,30] and the LSU rDNA D1–D2 or D1–D3 of *P. australis* [31], ‘*P. calliantha*’, *P. multistriata*, and *P. subpacifica* (registered as *P.* cf. *heimii* in the DDBJ/EMBL/GenBank) [10], and ‘*P. turgidula*’ [32]. We also determined the LSU rDNA D1–D3 sequences of representative isolates to compare them with previous sequences from New Zealand. Genotype screening of the 130 isolates based on the ITS region and/or LSU rDNA D1–D3 reconfirmed the presence of ten previously reported species [*P. americana*, *P. australis*, *P. calliantha*, *P. delicatissima*, *P. fraudulenta*, *P. multiseries*, *P. multistriata*, *P. plurisecta*, *P. pungens*, and *P.* (cf.) *subpacifica*]. In addition, presence of four species (*P. arenysensis*, *P. cuspidata*, *P. galaxiae*, and *P. hasleana*), previously unreported from New Zealand’s coastal waters, were confirmed with one to seven strains of each newly reported species. This result suggests that establishing a larger number of isolates for genotype screening would enhance research efforts for the identification of rare species. Therefore, in order to conduct comprehensive genotype screening globally, a large number of isolates are required to clarify *Pseudo-nitzschia* species diversity and geographical distribution, including the locations of rare species.

The clonal isolates of *P. delicatissima* belonged to two (I and II) of the three previously reported subclades (I, II, and III; [14]). Although previous studies in New Zealand [31,32,33] reported the existence of this species based on the results of morphological characterisation and/or *P. delicatissima*-specific Fluorescence In Situ Hybridization (FISH) assays, the sequences of wild cells or isolates of this species were not determined. Thus, the subclade information for the *P. delicatissima* specimens reported could not be confirmed. Regarding this species, Rhodes et al. (1998a, 1998b) [31,32] reported that strain CAWB12 from New Zealand showed morphological features of *P. turgidula*, while it was positively labelled with the *P. delicatissima*-specific FISH assay. Rhodes et al. (1998a) [32] also determined a sequence of the LSU rDNA D1–D2 for that strain and reported that the sequence was identical to that of *P. delicatissima* strain CV3, assigned as *P. delicatissima* subclade I in the present study. This molecular phylogenetic result suggests that the ‘*P. turgidula*’ strain CAWB12 belongs to *P. delicatissima* subclade I. Considering this finding, along with the present study confirming *P. delicatissima* subclades I and II, there is a possibility that the species previously reported as ‘*P. delicatissima*’ from New Zealand could correspond to *P. delicatissima* subclade II. Unfortunately, as the ‘*P. delicatissima*’ strains reported previously no longer exist, this could not be confirmed. To resolve this issue, detailed morphological observations for the strains of *P. delicatissima* subclades I and II and comparisons of their morphological features with those previously reported for the strains of ‘*P. delicatissima*’ and ‘*P. turgidula*’ are needed in the future.

In the molecular phylogenetic trees, a discrepancy in subclade separation of *P. delicatissima* from New Zealand between the ITS region and LSU rDNA D1–D3 was found. The *P. delicatissima* subclade I strains were clustered in an identical position in both trees. However, the subclade II strains (CAWB143 and CAWB144), assigned based on the ITS region tree, did not cluster with another strain of subclade II (strain PLY1St.46A) but clustered with a strain of subclade III (strain 318-5) in the LSU rDNA D1–D3 tree. A similar discrepancy between the ITS region and LSU rDNA D1–D3 was also reported in *P. galaxiae* [34]. Despite that the ITS region sequence of *P. galaxiae* strain 818-A2G was similar to that of *P. galaxiae* clade II strains (e.g., 818-A1G and Mex23), the LSU rDNA D1–D3 sequence of strain 818-A2G clustered with those of clade I. Recently, Kim et al. (2020) [35] conducted a mating experiment using *P. pungens* clades I–III strains and reported that successful mating results were observed in two combinations of clades I/II and clades I/III. The authors also reported that offspring strains whose parents were clades I and III strains had ITS2 copies of clade I, clade III, and hybrid type. This finding revealed the possibility of mating between different genetic clades in the same species. More information on the possibility of mating between *P. delicatissima* subclades I, II, and III is required. Although the subclade II strains established in this study had the sequence of subclade III in the LSU rDNA D1–D3 as discussed above, no *P. delicatissima* subclade III strains have been established from New Zealand so far. There is a need to further investigate the diversity of *P. delicatissima* in New Zealand to reveal whether subclade III is widely distributed. It should also be noted that the ITS region sequences of four New Zealand strains of *P. delicatissima* subclade II were slightly different from those of the others belonging to the same subclade. This difference may relate to the discrepancy in the LSU rDNA D1–D3. Further genetic investigations of the New Zealand genotype of *P. delicatissima* subclade II need to be conducted in the future.

The present study successfully established clonal strains genetically assigned as closely related species, *P. cuspidata* clade Ia and *P. plurisecta*, from New Zealand. So far, Rhodes et al. (2013) [10] established a ‘*P. cuspidata*’ strain from New Zealand and reported that the strain showed morphological features of *Pseudo-nitzschia* sp. strain Hobart5 [36]. Strain Hobart5 was then transferred to a newly described species, *P. plurisecta* in 2013 [37]. Based on this background, the present study updated the identification of the previous New Zealand ‘*P. cuspidata*’ strain to *P. plurisecta*, making it the first record of *P. cuspidata* in New Zealand. The ITS phylogeny suggested that *P. cuspidata* strains were divided into at least two clades (I and II). A couple of other *P. cuspidata* strains (Tenerife8 and NWFSC194) were clustered together with *P. pseudodelicatissima* strains. Rivera-Vilarelle et al. (2018) [38] established *P. cuspidata* strains from Mexico (e.g., strains Ps116 and Ps142) and described them as a variety of *P. cuspidata*, *P. cuspidata* var. *manzanillensis*. These strains belonged to clade Ib and clustered with two Chinese *P. cuspidata* strains (MC3041 and MC3027) reported by Huang et al. (2019) [39]. However, in the publication from Huang et al. (2019) [39] there was no detailed morphological information on these Chinese strains, which is needed to determine if the strains morphologically resemble *P. cuspidata* var. *manzanillensis*. In the phylogeny, *P. cuspidata* clade II was a sister to a cluster comprised of *P. pseudodelicatissima* strains and two *P. cuspidata* strains (Tenerife8 and NWFSC194) rather than *P. cuspidata* clade I. Furthermore, the genetic diversity within *P. cuspidata* clade II was similar to that between *P. cuspidata* clades Ia and Ib, suggesting clade II was comprised of several genotypes. These results suggest the necessity to assess taxonomic status of *P. cuspidata* clade II in the future. Recently, Ajani et al. (2021) [18] re-assigned Australian *P. cuspidata* clade II strains as *P.* cf. *cuspidata*. It should be noted that the separation of the cryptic *P. cuspidata* and *P. pseudodelicatissima* remains unresolved and should be clarified in the future, as discussed in previous studies [26].

This study presents the first record of *P. arenysensis* in New Zealand. *Pseudo-nitzschia arenysensis* was assigned previously as *P. delicatissima* clade B [40] or *P. delicatissima* del1 [27] and then described as a new species in 2009 [41]. Quijano-Scheggia et al. (2009) [41] reported that *P. arenysensis* was morphologically indistinct from *P. delicatissima* but differed in physiological parameters, such as the temperature, needed to trigger reproductive sexual, growth rates, and rate of cell size reduction. Therefore, the reason that *P. arenysensis* has not previously been recorded from New Zealand may be due to this species’ morphological resemblance to *P. delicatissima* and/or that *P. arenysensis* is a rare species as discussed above.

This study is also the first record of *P. galaxiae* and *P. hasleana* in New Zealand. *P. galaxiae* was described from Mexico by Lundholm and Moestrup (2002) [42]. Later, McDonald et al. (2007) [19] conducted clone library sequencing of the LSU rDNA for field samples and isolates from Italy. The authors recognised five *P. galaxiae* clades (I–V) and revealed that clades I–III corresponded to those cell sizes based on observations using those clonal cultures [19]. Unfortunately, these sequences could not be included in our analyses as they were short reads (partial LSU rDNA D1–D2) but several sequences from clade I (strain Sydney4), clade II (strains Mex23 and SM26), and clade III (strain SM10) reported previously (Figure 2 in [19]) could be included. Although clades I and II (in the LSU rDNA) corresponded to clades A and B (in the ITS region), respectively, the correspondence of clade III was unclear in the ITS region phylogeny. Thus, the present study coded the *P. galaxiae* clades as A–C, instead of clades I–V, in the ITS region phylogeny. It would be desirable to determine the ITS region sequences of clades IV and V and the LSU rDNA sequences of clade C to clarify the clades correspondence and reveal the diversity of this species.

*Pseudo-nitzschia hasleana* was described from USA by Lundholm et al. (2012) [43]. Although the morphological features of this species were similar to other closely related species (e.g., *P. calliantha* and *P. mannii*), the molecular phylogenetic position of *P. hasleana* was separated from the other related species [43]. The present study found the previously reported genotype of *P. hasleana* (assigned as clade I) and discovered another genotype (assigned as clade II) closely relating to clade I. The comparison of the average *p* distance of the ITS region between these clades (I and II) and other species, having clades/subclades, revealed that the value between *P. hasleana* clades I and II was the same as that between *P. pungens* clades I and II. Moreover, the average value between *P. hasleana* clades I and II was smaller than the values calculated between other species combinations. This comparison suggests that *P. hasleana* clade II may belong to *P. hasleana* rather than the other species or new species.

Concerning *P. hasleana*, Rhodes et al. (2013) [10] reported a sequence of the LSU rDNA D1–D3 of ‘*P. calliantha*’ strain CAWB114, whose morphology was similar to that of *P. calliantha*, from New Zealand. The LSU rDNA D1–D3 phylogeny in the present study demonstrated that ‘*P. calliantha*’ strain CAWB114 was clustered together with *P. hasleana* clade II. The average *p* distance comparisons suggest that ‘*P. calliantha*’ strain CAWB114 is closer to *P. hasleana* than to *P. calliantha*. The comparisons also suggest that ‘*P. calliantha*’ would be a new clade of *P. hasleana* or an undescribed species. Unfortunately, ‘*P. calliantha*’ strain CAWB114 died, and we could not compare the ITS region sequences between *P. hasleana* clades I and II and strain CAWB114. The establishment of new isolates of ‘*P. calliantha*’ is, therefore, required. In the future, detailed morphological observations and comparisons of *P. hasleana* clades I and II and ‘*P. calliantha*’ strains are needed. It is also necessary to assess their mating potential and ITS2 secondary structure to confirm their taxonomic relationship.

One of the remaining issues of *Pseudo-nitzschia* classification is between *P. heimii* and *P. subpacifica*. Although these two species closely resemble each other morphologically, both species can be distinguished by comparing some morphological characters (i.e., density of fibulae, striae, and poroids) [10,44]. In 1996, Rhodes et al. (1998a, 1998b) [31,32] reported *P. heimii* based on results of the *P. heimii*-specific FISH assay from New Zealand. The authors, then, re-evaluated this species using isolates collected in 1998 and suggested that the correct morphological identification of this species in New Zealand is *P. subpacifica* [10]. At the same time, the authors also determined an LSU rDNA D1–D3 sequence of *P. subpacifica* (reported as *P.* cf. *heimii*) strain CAWB106 and reported it to have 99% identity to *P. heimii* and *P. subpacifica* sequences deposited in DDBJ/EMBL/GenBank so far [10]. Molecular phylogeny of the LSU rDNA D1–D3 for some strains established in the present study suggested that they were clustered together with the previously reported *P. subpacifica* strain CAWB106. Although detailed morphological observations of these newly established strains have not been conducted yet, the present study assigned these strains as *P.* cf. *subpacifica* rather than *P.* cf. *heimii* as a temporary assignment. Detailed morphological observations should be made in the future to identify whether these strains are in fact *P. subpacifica*.

Other than the 14 species found in the present study, previous New Zealand studies reported ‘*P. pseudodelicatissima*’ in 1996 [32] and ‘*P. caciantha*’ in 2005 [10] based on the results of the species-specific FISH assay and/or morphological observations. There were however no rDNA sequences determined for these species from New Zealand. After the report of Rhodes et al. (1998a) [32], *P. pseudodelicatissima* classification became confused as the number of reports of *Pseudo-nitzschia* increased [36], and many morphologically similar species were molecular phylogenetically divided into three groups of the ‘*P. pseudodelicatissima* complex’ [26]. Rhodes et al. (2013) [10] reported ‘*P. caciantha*’ strains by morphological identification from three sites in New Zealand. Subsequently, the strains from two of three sites were confirmed as *P. calliantha* based on sequences of the ITS region (Rhodes, unpublished data). Thus, the ‘*P. caciantha*’ strains from the two sites should be re-assigned as *P. calliantha*. The assignment of the strains from the remaining one site needs to be re-evaluated. Therefore, the previous reports of ‘*P. pseudodelicatissima*’ and ‘*P. caciantha*’ in New Zealand should be treated as tentative until the clonal isolates, showing rDNA sequences of each species, are established in the future.

As mentioned above, although morphological features of each *Pseudo-nitzschia* species/clade/subclade in New Zealand were not investigated in the present study, these could be important for distinguishing them from each other. The features will be investigated in future research.

### 3.2. Toxin Production of Pseudo-nitzschia Species in New Zealand

There are so far ten known analogues of DA and DA isomers, namely DA, epi-DA, iso-DAs A, B, C, D, E, F, G, and H. From these ten analogues, only DA is currently regulated within the international Codex standard 292-2008 [6] and the New Zealand Animal Products Act 1999 [7] at a maximum permissible level of 20 mg kg^−1^ in shellfish. Additional isomers have however been reported [8], but there is insufficient toxicity information to establish Toxicity Equivalency Factors (TEF) for the DA isomers [9]. Because DA can convert to epi-DA during storage [45,46], the acute reference dose is currently applied to the sum of both DA and epi-DA, assuming equal relative toxicity. Therefore, DA and epi-DA are under surveillance in New Zealand shellfish. The European Food Safety Authority CONTAM panel concluded in their 2009 scientific opinion that setting TEF values for the other DA isomers was not required as the iso-DAs typically occur at lower concentrations and are less toxic than DA [47]. As the DA isomers do not have unique differentiating features by MS/MS analysis, only peaks which were identified as having consistent retention time with reference standards are able to be identified, hindering the research into DA isomer production. In the absence of iso-DAs F–H reference material, purified standards, or a QC sample, these analogues were not included in the present study.

Domoic acid is the primary ASP-causing toxin, with multiple studies assessing DA production by *Pseudo-nitzschia*. In contrast, there are only a few studies which assess DA isomer production by *Pseudo-nitzschia* strains: iso-DAs A and B from *P. delicatissima*, *P. multiseries* [48], and *Pseudo-nitzschia* *seriata* [49], iso-DA C from *P. australis* [50,51] and *P. subcurvata* [52], and epi-DA and iso-DAs A and D from *P. plurisecta* [53]. This is the first study to screen, and report, iso-DA E production by *Pseudo-nitzschia* strains. In addition, this is the first report of large scale screening of *Pseudo-nitzschia* strains (73 in total representing 14 species) for the production of DA, epi-DA, iso-DAs A, B, C, D, and E. The production of DA and five of the DA isomers (epi-DA and iso-DAs A–D) was subsequently detected in strains of *P. australis* and *P. multiseries*. In addition, a previously unreported DA isomer from *Pseudo-nitzschia*, iso-DA E, was detected in four strains of *P. multiseries* and two strains of *P. australis* for the first time. Details of DA and DA isomers production by New Zealand *Pseudo-nitzschia* strains are discussed below.

Rhodes et al. (2013) [10] reviewed the previous studies reporting the DA production by New Zealand strains assessed using an immunoassay, and liquid chromatography-ultraviolet detection (LC-UV) and/or LC-MS/MS analytical chemistry techniques. The authors reported that the strains belonging to seven of the 12 reported species produced DA. The authors also pointed out that there were differences in DA production and related potential ASP risk among these reported species: potential ‘high ASP risk’ species (four species with maximum DA production ≥0.5 pg cell^−^^1^), potential ‘low ASP risk’ species (three species with maximum DA production ≤0.1 pg cell^−^^1^), and potential ‘no ASP risk’ species (five species with DA production not detected) [10]. Of these, one of the potential ‘high ASP risk’ species, *P. australis,* was reported as the primary DA producing species in New Zealand, with all strains of this species analysed to date producing DA at concentrations ranging from 0.05 to 2.20 pg cell^−^^1^ [12,31,32,33,50,54]. Wild cells of this species were reported to produce DA ranging from ‘not detected’ to 35.00 pg cell^−^^1^ [31]. The present study cultured strains of 14 *Pseudo-nitzschia* species following the culturing method reported by previous studies in New Zealand, using an f/2−Si medium to make the cultures stressed to produce DA and DA isomers under silicate limitation [10,54]. As a result, all nine *P. australis* strains analysed produced DA, and their DA cell quotas ranged from 0.004 to 2.02 pg cell^−^^1^. These results demonstrated that the DA cell quotas of the strains tested in the present study were similar to those of previous strains of the same species from New Zealand. On the other hand, the maximum DA cell quota of 35.00 pg cell^−^^1^ was reported so far from the wild *P. australis* cells as mentioned above, suggesting that the DA cell quota of the wild cells could be higher than those of cultured strains in some cases. In this regard, previous studies reported that abiotic (temperature, irradiance, salinity, pH/partial pressure of CO_2_, trace metals, inorganic and organic nitrogen, limitation of phosphorus and silicon, and those interactions) and biotic (bacteria and grazing copepods) factors enhance DA production of various *Pseudo-nitzschia* species [25,55,56]. Rhodes et al. (2004) [54] also reported enhanced DA production of a *P. australis* strain from New Zealand under increased trace metal (i.e., copper) conditions in f/2−Si medium. Thus, the highest DA cell quota from the wild *P. australis* cells reported previously [31] might be enhanced by one or many of the factors above. In the future, the DA production of *P. australis* strains from New Zealand will be examined under a variety of culture conditions other than the factors reported previously by Rhodes et al. (2004) [54]. Another factor might be a decrease in DA production for the cultured strains resulting from long-term culturing, as reviewed in Lelong et al. (2012) [55]. The present study conducted culturing and toxin analysis for cultures of *P. australis* strain G015Ps04 at three and seven months after the strain was established. The results showed that the DA cell quota of the ‘seven month old’ culture (0.004 pg cell^−^^1^) was almost ten times lower than that of the ‘three month old’ culture (0.05 pg cell^−^^1^). Although this hypothesis needs to be evaluated using multiple strains in the future, this factor could potentially be one of the reasons why the DA cell quota from the wild cells appeared high in some cases.

The second potential ‘high ASP risk’ species in New Zealand is *P. multiseries*. Rhodes et al. (1998a) [32] reported that one *P. multiseries* strain produced 0.80 pg cell^−^^1^ of DA. The present study established four strains of this species and found that all strains produced DA ranging from 0.47 to 7.20 pg cell^−^^1^. Although the lowest value was lower than that previously reported, the highest value was almost ten times higher than that previously reported. Considering this point, the difference in the DA cell quota might be due to the differences in DA productivity among the *P. multiseries* strains, similar to the results for *P. australis* strains above. It should be noted that all established strains of *P. australis* and *P. multiseries* tested so far produced DA, with the highest DA cell quota of the *P. multiseries* strains (7.20 pg cell^−^^1^) being greater than that of the *P. australis* strains (2.20 pg cell^−^^1^) in New Zealand.

The third and fourth potential ‘high ASP risk’ species in New Zealand are *P. multistriata* and *P. pungens*. Previous studies from New Zealand reported that some strains of these species produced DA, whereas others did not [12,32,33,50,54,57,58]. The present study revealed that all eight strains of *P. multistriata* and 13 strains of *P. pungens* tested did not produce quantifiable levels of DA, supporting the previous reports above. These results suggest that although *P. multistriata* (‘not detected’–1.60 pg cell^−^^1^) and *P. pungens* (‘not detected’–0.47 pg cell^−^^1^) were assigned as potential ‘high ASP risk’ species, their risk to ASP might be lower than the other two species, *P. australis* (0.004–2.20 pg cell^−^^1^) and *P. multiseries* (0.47–7.20 pg cell^−^^1^).

Rhodes et al. (2013) [10] assigned three species (*P. delicatissima*, *P. fraudulenta*, and ‘*P. pseudodelicatissima*’) as potential ‘low ASP risk’ species in New Zealand. Previous New Zealand studies reported DA production from three *P. delicatissima* strains (0.03–0.12 pg cell^−^^1^, two of them were reported as ‘*P. turgidula*’) [12,32], only one of three *P. fraudulenta* strains (0.03 pg cell^−^^1^) [32,50,54], and one ‘*P. pseudodelicatissima*’ strain (0.12 pg cell^−^^1^) [32]. The present study tested DA production in *P. delicatissima* subclades I (five strains) and II (four strains) and *P. fraudulenta* (five strains), and revealed these strains did not produce detectable levels of DA. This result supports the recommendation that these species should be kept as potential ‘low ASP risk’ species in New Zealand.

Rhodes et al. (2013) [10] assigned five species [*P. americana*, ‘*P. caciantha*’, *P. calliantha*, *P. plurisecta* (formerly reported as *P. cuspidata* Hobart-5 type), and *P. subpacifica* (formerly reported as *P.* (cf.) *heimii*)] as potential ‘no ASP risk’ species in New Zealand as previous strains of these species analysed did not produce detectable levels of DA [58]. The present study tested DA production in *P. americana* (four strains), *P. calliantha* (three strains), *P. plurisecta* (one strain), and *P.* cf. *subpacifica* (four strains), confirming the previous report that these species in New Zealand do not produce detectable levels of DA. Regarding the four newly recorded species (*P. arenysensis*, *P. cuspidata*, *P. galaxiae*, and *P. hasleana*), previous studies from other countries reported that *P. arenysensis* strains tested did not produce DA, while some strains of *P. cuspidata*, *P. galaxiae*, and *P. hasleana* did [25,28,39,59]. The present study tested DA production in *P. arenysensis* (six strains), *P. cuspidata* (one strain), *P. galaxiae* (two strains), and *P. hasleana* (four strains), revealing none of the strains tested produced DA above the limit of detection. As an exception, one *P. cuspidata* strain, CAWB141, established in the present study did not produce DA on day nine, however there were trace levels (the value between LoD of 0.00008 and LLoQ of 0.0008 pg cell^−^^1^) on day 43. Although this strain produced a trace level of DA, its cell quota was very low compared with those of potential ‘high and low ASP risk’ species. Thus, the present study tentatively assigned *P. cuspidata* as potential ‘no ASP risk’ species. It should be noted that more *P. cuspidata* strains need to be established and analysed for toxin production to determine if this species should remain assigned as a potential ‘no ASP risk’ species in New Zealand moving forward. In the case of *P. cuspidata* strains from New South Wales, Australia, the strains produced DA ranging from ‘not detected’ to 24.5 pg cell^−^^1^ [59]. In the ITS region tree, one of the Australian strains [strain MER, re-assigned as *P.* cf. *cuspidata* by Ajani et al. (2021) [18]] belonged to *P. cuspidata* clade II, which differs from clade I to which the New Zealand strain CAWB141 belonged. In addition, in the case of *P. cuspidata* strains from Barkley Sound, Canada and Washington state, USA, isolated from single-species blooms of *Pseudo-nitzschia* that produced DA (up to 63 pg cell^−1^), the strains tested produced DA [60]. In the ITS region, one of these *P. cuspidata* strains (strain NWFSC194) belonged to a cluster comprised of *P. cuspidata* and *P. pseudodelicatissima* strains, which differs from clade I to which the New Zealand strain CAWB141 belonged. It is, therefore, suggested that the toxin production of each *P. cuspidata* clade and cluster may differ. The diversity and toxin production of additional *P. cuspidata* strains from various geographic locations need to be assessed in the future. As discussed above, some strains of the nine potential ‘no ASP risk’ species from other countries produced DA, except for *P. americana* and *P. arenysensis* [25]. However, none of the strains of these species tested from New Zealand produced DA. This difference might be caused by the clade/subclade/genotype/population-level differences of the tested strains among those localities, occurrence seasons, or bloom years. Although the result of the present study supports the recommendation that the nine species should be kept as potential ‘no ASP risk’ species, at least in New Zealand, continuous assessment for the toxin production of clonal isolates of these species is needed to reassess their assignations in the future.

Apart from DA, the present study detected all, or a combination of, the tested DA isomers (epi-DA and iso-DA A–E) from all *P. australis* and *P. multiseries* strains analysed. Recently, Olesen et al. (2021) [52] reported relative amounts of DA and iso-DA C in three *P. subcurvata* strains. The authors reported that the proportion of DA tended to be lower than that of iso-DA C (DA:iso-DA C = 34–49%:51–66%) [52]. Unlike the results for *P. subcurvata* strains [52], the present study found that the proportions of DA of *P. australis* and *P. multiseries* strains tended to be higher than those of iso-DA C [*P. australis*, DA:iso-DA C = 44.4–91.5%:4.8–54.1%; *P. multiseries*, 93.9–96.5%:2.3–3.1%], suggesting that the ratio of DA to DA isomers varies among species. In addition to this, Rhodes et al. (2004, 2006) [54,61] reported that increased trace metal conditions (e.g., zinc, cobalt, copper, or selenium) in f/2−Si medium enhanced the iso-DA C production in a New Zealand *P. australis* strain. The assessment on the enhanced DA and DA isomers production for *P. multiseries* strains has not been investigated yet. It is, therefore, necessary to examine the enhancements in the production of DA and DA isomers using other *P. australis* and *P. multiseries* strains in the future.

### 3.3. Distribution of Pseudo-nitzschia Species with Potential ‘High, Low, or No ASP Risk’ in New Zealand

Determining *Pseudo-nitzschia* species diversity and distribution, along with their respective DA and DA isomers cell quota, is essential in assessing the ASP risk in New Zealand. In 2011, Rhodes et al. (2013) [10] reviewed previous research on this genus in New Zealand, conducted over 20 years, and summarised the distribution of each species (Table 1 in [10]). As the status of *Pseudo-nitzschia* research has not been updated since this study, we aimed to refresh this information. The comparisons of distribution patterns of the 16 species, based on the analysis for the clonal isolates, recorded from New Zealand to date, suggest that most of the potential ‘high and low ASP risk’ species were widely distributed in the subtropical and temperate zones of New Zealand, compared to the potential ‘no ASP risk’ species which had restricted distributions. Additionally, the most critical species (*P. australis* and *P. multiseries*) for ASP risk assessment were recorded from both zones. Continuous monitoring of the potential ‘high ASP risk’ species, especially *P. australis* and *P. multiseries*, is therefore necessary to minimise the risk of ASP in New Zealand. As mentioned in previous study [56], it should be noted that bias can be introduced in these distribution records because the data were generated only from isolates that survived the culturing process employed in the previous and present studies in New Zealand. These isolates also might be biased by the collected field populations, which might have some factors influencing success in culture (e.g., seasonality and cell health).

Three of the four potential ‘high ASP risk’ species (*P. australis*, *P. multiseries*, and *P. pungens*) have larger cells compared with the other 13 recorded species. Another potential ‘high ASP risk’ species, *P. multistriata*, has smaller cells than the three species above, and it may be distinguishable due to its sigmoidal cell shape compared with the other 12 smaller recorded species from the coastal waters [62]. However, as Lelong et al. (2012) [55] discussed, precise determination of *Pseudo-nitzschia* species identity by light microscopy is difficult, if not impossible, because most of the frustule morphometrics needed for species determination are visible only by scanning or transmission electron microscopy. One of the solutions for this problem is the use of molecular tools, such as a real-time PCR or FISH assays, that enables morphologically similar species in field samples to be reliably differentiated for monitoring *Pseudo-nitzschia*. Real-time PCR assay uses a primer set or a primer/probe set, that reacts to each target species’ specific DNA regions. This method can, therefore, specifically detect and enumerate each target species, and is expected to be a powerful tool for revealing detailed distribution patterns and for monitoring each target species’ cell numbers and dynamics. These real-time PCR assays have been developed for several *Pseudo-nitzschia* species to date, specifically looking for: the genus *Pseudo-nitzschia* [63,64]; *Pseudo-nitzschia* *brasiliana*, *P. calliantha*, *P. delicatissima*, *P. arenysensis*, *P. fraudulenta*, *P. galaxiae*, *P. multistriata*, and *P. pungens* [65]; and *P. pungens* clades I/II and *P. pungens* clade III [30]. From the four potential ‘high ASP risk’ species in New Zealand, the assay has not been developed for *P. multiseries*. An assay targeting this species is currently under development (Bowers et al. unpublished data). It is expected that this method will be used to examine the detailed distribution and dynamics of each potential ‘high ASP risk’ species, and will be used to conduct a detailed ASP risk assessment for New Zealand in the future.

## 4. Materials and Methods

### 4.1. Sampling and Establishment of Clonal Isolates

As part of the New Zealand Marine Phytoplankton Monitoring Programme, 33 field seawater samples were collected using mainly a depth integrated hose sampling method, from 22 sampling sites (0–15 m depths) of New Zealand coastal waters, the south Pacific Ocean, between 2018 and 2020. Duplicate 100 mL samples were collected each time and Lugol’s iodine solution was added to one of the two bottles in order to fix the microorganisms for future microscopic investigations, and the other bottle was used for cell isolations, and subsequent culturing. These bottles were immediately transported to the Micro-algae Laboratory at the Cawthron Institute, Nelson, New Zealand. The sampling locations comprised four sites from the subtropical zone (North part of the North Island) and 18 sites from the temperate zone (South part of North Island, South Island, and Stewart Island) (Figure 4). Details, including sampling site code, and the latitude and longitude coordinates of each site, are shown in Table S1.

At Cawthron Institute, the presence of *Pseudo-nitzschia* in the fixed samples was investigated using Utermöhl chambers and inverted light microscopes (CK-40 or CK-41; Olympus, Tokyo, Japan). Once the presence of *Pseudo-nitzschia* cells was confirmed, the replicate live seawater sample collected at the same time was used for cell isolation. The live sample was transferred to a 6-well flat-bottom cell culture plate (Costar 3516; Corning, NY, USA) and individual *Pseudo-nitzschia* cells or chains were isolated and washed with three drops of sterile f/2 medium [66], containing Na_2_SiO_3_·5H_2_O at 59 µmol L^−1^ in the present study instead of Na_2_SiO_3_·9H_2_O at 54–107 µmol L^−1^, on a glass plate with a drawn-out Pasteur micropipette, using an inverted microscope (CK-2; Olympus). The sterile medium was prepared using autoclaved seawater (salinity adjusted to 33) and membrane filtration (GSWG047S6; 0.22 µm, 47 mm, Millipore, MA, USA). Each isolated cell or chain was then transferred to a separate well of a 24-well flat-bottom cell culture plate (Costar 3524; Corning) filled with 1 mL of the sterile f/2 medium. The 24-well plates containing the isolated cells were kept at 18 ± 1 °C and 40–90 μmol photons m^−2^ s^−1^ with a photoperiod of 12:12 h L:D. After a sufficient cell density was achieved, the culture media, including the cells in each well, were transferred into a clear polystyrene container (LBS32002NX; LabServ, Auckland, New Zealand) containing 20 mL of fresh sterile f/2 medium. The established clonal cultures were maintained under the culturing conditions described above and inoculated every ten days. Details of the clonal strains are shown in Table S2. A total of 30 representative strains established in the present study (CAWB127–CAWB156) are deposited and maintained in the Cawthron Institute Culture Collection of Microalgae (CICCM; Nelson, New Zealand).

### 4.2. Molecular Phylogenetic Characterisation

#### 4.2.1. DNA Extraction, PCR, and Sequencing

Cultures of the clonal *Pseudo-nitzschia* isolates were harvested at stationary phase, around seven days after inoculation, in 1.5 mL screw-cap microcentrifuge tubes (Labcon, CA, USA) by centrifugation (at 9000× *g* for 2 min, MiniSpin plus; Eppendorf, Hamburg, Germany). Genomic DNA of isolates was extracted using the DNeasy PowerSoil kit (Qiagen, Hilden, Germany) according to the manufacturer’s protocol or by the chelex-based DNA isolation method [67]. Briefly, the cell pellets were dissolved in 200 μL of 10% (*w* *v*^−1^) solution of Chelex 100 Resin (143-2832; Bio-Rad, CA, USA) in a 1.5 mL tube (Labcon) and incubated at 95 °C for 20 min with thorough mixing every 10 min. The tube was then centrifuged at 9000× *g* for 2 min (Centrifuge 5430; Eppendorf). The supernatant containing genomic DNA was used as a template for PCR amplification below.

The rDNA, specifically, the ITS region and LSU rDNA D1–D3 was amplified by PCR. The PCR was performed using 25 µL reaction volumes as follows: nuclease-free water (AM9937; Ambion, CA, USA); 12.5 µL of MyTaq Red Mix, 2 × (Bioline, London, UK); each primer (0.4 µM as a final concentration) as below; non-acetylated bovine serum albumin (BSA, 16 ng; Sigma-Aldrich, Auckland, New Zealand), and template genomic DNA (approximately 5–100 ng) extracted as above. The ITS region was amplified using the primers: 4618F (forward, 5′-GTA GGT GAA CCT GCA GAA GGA TCA-3′) and LSU1R (reverse, 5′-ATA TGC TTA AAT TCA GCG GGT-3′) [68]. The LSU rDNA D1–D3 was amplified using the primers: D1R (forward, 5′-ACC CGC TGA ATT TAA GCA TA-3′) [21] and D3B (reverse, 5′-TCG GAG GGA ACC AGC TAC TA-3′) [69]. PCR was run using a thermocycler (Mastercycler nexus gradient; Eppendorf), and PCR cycling conditions were 98 °C for 4 min; 35 cycles of 95 °C for 30 s, 60 °C for 30 s, and 72 °C for 60 s; and 72 °C for 10 min. For identifying positive bands with a known standard, the PCR products were run on a 1.5% (*w* *v*^−1^) agarose gel, stained with RedSafe Nucleic Acid Staining Solution (iNtRON Biotechnology, Seoul, Korea), and viewed under a UV light.

The PCR products were purified using NucleoSpin Gel and PCR Clean-up kit (Macherey-Nagel, Düren, Germany) according to the manufacturer’s protocol and sequenced using the PCR primers by Genetic Analysis Services, University of Otago, Dunedin, New Zealand or Macrogen, Seoul, South Korea. Forward and/or reverse reads were edited and assembled using Geneious 8.0.5 (Biomatters, Auckland, New Zealand). Representative sequences were deposited in DDBJ (DDBJ accession numbers: LC636495–LC636593; the primer regions located at both ends of the sequences were excluded from the deposited sequences) (Table S2).

#### 4.2.2. Molecular Phylogenetic Analyses

To estimate the molecular phylogenetic positions of New Zealand *Pseudo-nitzschia* based on the ITS region sequences, 190 publicly available sequences of 52 *Pseudo-nitzschia* species and two sequences of *Cylindrotheca closterium* as an outgroup were obtained from the DDBJ/EMBL/GenBank database. Compared with the LSU rDNA D1–D3 data set below, sequences of *P. linea* were not included in the ITS region data set because these sequences have not yet been determined. A total of 61 representative sequences of the ITS region of New Zealand *Pseudo-nitzschia* strains were aligned with the reference sequences above using ClustalW [70] in Geneious 8.0.5 (Biomatters). In this data set, the 5′ and 3′ ends were manually aligned to truncate, and both ends were refined. The final alignment consisted of 253 sequences of 1185 positions, including gaps [the alignment site corresponded to the 156–761 bp site of a sequence of *P. seriata* strain Lynæs6 (DDBJ/EMBL/GenBank accession number: DQ062663)]. Phylogenetic analysis was conducted using the maximum likelihood (ML) method by MEGA X [71]. To determine the best DNA model, all positions with less than 10% site coverage were eliminated (partial deletion option available in MEGA X), and the final data set was a total of 809 positions. The best-fit model of nucleotide substitution selected based on the Akaike information criterion (AIC) (find best DNA/protein models option available in MEGA X) was found to be the GTR + G + I model. Bootstrap analysis of 1000 replicates was performed to examine the robustness of the clades. For the phylogenetic analysis, all positions were treated using the same parameters as those used for the DNA model analysis described above. Phylogenetic analysis was also conducted using the Bayesian inference (BI) method using MrBayes 3.1.2 [72] to estimate the posterior probability (pp) distribution using Metropolis-coupled Markov chain Monte Carlo (MCMCMC) [73]. For BI analysis, MrModeltest 2 [74] was used to determine the best-fit model of nucleotide substitution using PAUP 4.0b10 (Sinauer Associates, MA, USA). The best-fit model, according to the AIC, was the GTR + G + I model. The analysis was performed using four chains with temperature set 0.2. The analysis was performed with seven million generations, and the trees were sampled every 100 generations. For increasing the probability of chain convergence, 16,000 trees were sampled after average standard deviation of split frequencies (ASDSF) was below 0.01 to calculate the pp.

To construct the LSU rDNA D1–D3 molecular phylogeny, 139 publicly available sequences of 47 *Pseudo-nitzschia* species and two sequences of *Cylindrotheca closterium* as an outgroup were obtained from the DDBJ/EMBL/GenBank database. Compared with the ITS region data set above, sequences of six species (*P. bucculenta*, *P. hainanensis*, *P. obtusa*, *P. taiwanensis*, *P. uniseriata*, and *P. yuensis*) and three clade/subclades (*P. cuspidata* clade Ib, *P. decipiens* subclade A, and *P. galaxiae* clade C) were not included in the LSU rDNA D1–D3 data set because these sequences have not yet been determined. A total of 38 representative sequences of the LSU rDNA D1–D3 of New Zealand *Pseudo-nitzschia* strains were aligned with the reference sequences above using ClustalW, and both ends were refined following the method as described above. The final alignment consisted of 179 sequences of 710 positions for the LSU rDNA D1–D3 data set [the alignment site corresponded to the 74–729 bp site of a sequence of *P. seriata* strain Lynæs8 (DDBJ/EMBL/GenBank accession number: AF417653)]. Phylogenetic analysis was conducted using the ML method with the best-fit model (GTR + G + I model), following the procedures as above. The final data set was a total of 660 positions. Phylogenetic analysis was also conducted using the BI method with the best-fit model (GTR + G + I model), following the procedures as above. The analysis was performed with five million generations, and 19000 trees were sampled after ASDSF was below 0.01 to calculate the pp.

#### 4.2.3. Sequence Analysis

Sequence analysis for the ITS region and the LSU rDNA D1–D3 was conducted by calculating the *p* distance of selected combinations within and between *Pseudo-nitzschia* species/clades/subclades using the uncorrected genetic distance (UGD) model (*p*-distance model available in MEGA 7; [75]). The alignments used for the phylogenetic analyses above were used. All positions with less than 10% site coverage were eliminated (partial deletion option available in MEGA 7). The number of positions used for the final data sets is shown in Tables S3 and S4.

### 4.3. Toxin Analysis

#### 4.3.1. Culturing, Harvesting, and Toxin Extraction

To enable instrumental analysis (LC-MS/MS; Section 4.3.2) of DA and DA isomers (epi-DA, iso-DAs A, B, C, D, and E) production, 73 representative strains of the 14 *Pseudo-nitzschia* species (1–13 strains per species) genetically identified were cultured. These representative strains of each species were selected from each sampling site if multiple strains were established from multiple sites. Briefly, 7 mL of the strains cultured in f/2 medium for seven days (as described above) were inoculated into two clear polystyrene containers (LBS32002NX; LabServ), each containing 30 mL of f/2 minus Na_2_SiO_3_·5H_2_O medium (f/2−Si medium) (approximately 20% inoculum) and cultured for four or five days. The use of f/2−Si medium stresses the cultures into producing DA and DA isomers by culturing under silicate limitation. Subsequently, approximately 7 mL of the cultures were inoculated into four containers containing 30 mL of f/2 or f/2−Si media, or K medium (not containing Si) [76] (approximately 20% inoculum) for an additional 8–13 and/or 43 d (Table S5). The 10 mL of media containing the cultured cells, that had sunk to the bottom of the four containers, was removed using a micropipette and pooled into a 50 mL centrifuge tube (430829; Corning). A 100 µL aliquot of each pooled cultures was transferred into a 1.5 mL screw-cap microcentrifuge tube (Labcon) containing 890 µL of sterile seawater and 10 µL of Lugol’s iodine solution [final concentration of Lugol’s iodine solution was 1% (*v*/*v*)]. The cell counts for the fixed samples were conducted in triplicate drops on a boundary slide glass (S6113; Matsunami Glass, Osaka, Japan) using an inverted microscope (CK-2; Olympus). The 50 mL tube containing the pooled culture was centrifuged at 3214× *g* for 40 min (Centrifuge 5810 R; Eppendorf), the supernatants were discarded, and resultant pellets were stored at −20 °C until the toxins were extracted.

For the toxin extraction, 20% aq. acetonitrile (MeCN; *v* *v*^−1^) (A955-4, Optima LC-MS Grade; Thermo Fisher Scientific, Waltham, MA, USA) was added to the 50 mL centrifuge tube containing the pellet, at a ratio of 1 mL per ≤ 2 × 10^6^ cells, followed by sonication (10 min) using an ultrasonic bath (XUBA3, 35 W, 44 kHz; Grant Instruments, Cambridge, UK). The tubes containing the disrupted cells were centrifuged at 3214× *g* for 5 min at 4 °C (Centrifuge 5810 R; Eppendorf). The supernatants were transferred into 15 mL centrifuge tubes (339650; Thermo Fisher Scientific, MA, USA) and stored at −20 °C overnight. The tubes were then centrifuged again at 3214× *g* for 5 min at 4 °C (Centrifuge 5810 R; Eppendorf) to afford a clear supernatant, and finally 1 mL was transferred into a 2 mL glass autosampler vial (AR0-3910-13; Phenomenex, CA, USA) and stored at −20 °C until LC-MS/MS analysis.

#### 4.3.2. Instrument Analysis

Calibration was performed using a dilution of CRM-DA-g, certified reference material from the National Research Council of Canada (NRC; Halifax, Canada), which contains certified concentration of DA + epi-DA, and uncertified concentrations of iso-DAs A, D and E. A well-characterised reference material of iso-DA C obtained from Cawthron Natural Compounds (CNC; Cawthron Institute) was used to quantitate iso-DA C. A sample of iso-DA B obtained from CNC (Cawthron Institute) was used to confirm retention times and ion ratios for qualitative presence/absence analysis. A mussel certified reference material ASP-Mus-d contaminated with DA and DA isomers (epi-DA and iso-DAs A, D, and E) was obtained from NRC for quality control and used to obtain reference spectroscopic data.

Screening of DA and DA isomers was performed on a Waters Xevo TQ-S triple quadrupole mass spectrometer coupled to an Acquity I-Class UPLC with flow-through needle sample manager (LC-MS/MS; Waters, Milford, MA, USA), and was based on the LC-MS/MS lipophilic toxins method published by McNabb et al. (2005) [77]. Chromatographic separation used a Waters Acquity BEH Shield RP18 column (1.7 µm, 130 Å, 50 × 2.1 mm) held at 40 °C. The target analytes were eluted at 0.5 mL min^−1^ with (A) 5% aq. MeCN and (B) 95% aq. MeCN mobile phases, each containing 50 mM formic acid and 2.53 mM ammonium hydroxide. Initial conditions were 0% B and held for 0.2 min, then linearly increased to 15% B over 0.3 min, to 30% B over 0.5 min, to 80% B over 4 min then immediately increased to 100% B and held for 1.5 min before immediately returning to 0% B and holding for 0.5 min to re-equilibrate. The autosampler chamber was maintained at 10 °C and the injection volume was 1 µL. The mass spectrometer used an electrospray ionisation source operated in positive ion mode. Other settings were capillary voltage 2 kV, cone voltage 50 V, source offset 50 V, source temperature 150 °C, cone gas flow rate 150 L h^−1^, desolvation temperature 600 °C, desolvation gas flow rate 1000 L h^−1^, nebuliser gas flow 7 bar and the collision cell was operated with 0.15 mL min^−1^ argon. Multiple Reaction Monitoring (MRM) transitions for DA and DA isomers were *m*/*z* 312.2 > 266.2 (quantitation) and *m*/*z* 312.2 > 161.1 (confirmation), with collision energies of 16 and 25 eV, respectively. Both MRM transitions had a dwell time of 100 ms. The typical limit of detection (LoD) and lower limit of quantitation (LLoQ) of total of DA + DA isomers were 0.0005 and 0.0025 pg cell^−1^ (1 and 5 ng mL^−1^), respectively.

In addition, the screening of DA and DA isomers production was performed using LC-UV. This utilized a Waters Acquity I-Class UPLC with flow-through needle sample manager coupled to a photo diode array (PDA) detector (Waters, Milford, MA, USA) to confirm DA isomers identification. A Supelco Titan C18 column (1.9 µm, 80, 100 × 2.1 mm; Merck, Darmstadt, Germany), held at 40 °C, was used in conjunction with 17% aq. MeCN mobile phase, containing 0.1% trifluoroacetic acid. Isocratic elution was performed at a flow rate of 0.25 mL min^−^^1^. PDA detection was performed with a range of 190–350 nm, at a resolution of 1.2 nm, a sampling rate of 20 points per second and a time constant of 0.1 s. Domoic acid and epi-DA were analysed at 242 nm, and iso-DA C was analysed at 220 nm. The autosampler chamber was maintained at 4 °C and the injection volume was 1 µL. The typical LoD and LLoQ for DA + epi-DA were 0.025 and 0.075 pg cell^−^^1^ (50 and 150 ng mL^−^^1^), respectively.

Quantitative analysis of DA and DA isomers production, for the positive extracts screened by the LC-MS/MS or LC-UV techniques described above, was performed using LC-MS/MS on a Sciex 6500 + QTRAP tandem quadrupole mass spectrometer coupled to an ExionLC liquid chromatography system (Sciex, Framingham, MA, USA). A Biozen XB-C18 superficially porous column (1.7 µm, 100Å, 150 × 2.1 mm), held at 35 °C, (Phenomenex, Torrance, CA, USA) was used with (A) water and (B) MeCN mobile phases, each containing 0.1% formic acid. Initial conditions were 5% B at 0.3 mL min^−1^ linearly increasing to 15% B over 12.5 min, then increasing to 90% B over 2.5 min, held at 90% B for 1.5 min then returned to 5% B over 0.5 min and held to re-equilibrate for 3 min. Total run time was 20 min. The autosampler chamber was maintained at 4 °C and the injection volume was 1 µL. Pump compressibility compensation was enabled with pump A at 0.45/GPa and pump B at 1.20/GPa. Electrospray ionisation was performed in positive ion mode with curtain gas at 30, collision gas at high, ionisation voltage at 5500 V, temperature at 500 °C, ion source gas 1 at 50, and ion source gas 2 at 55. Declustering potential was set at 35, entrance potential at 9, and collision cell exit potential at 25. The quantitation MRM transition (*m*/*z* 312.2 > 266.2) was acquired with a collision energy of 21, and confirmation MRM transition (*m*/*z* 312.2 > 161.1) was acquired with a collision energy of 33 with a total 0.4 s cycle time. The typical LOD and LLoQ of each DA and DA isomers, except for iso-DA B, were 0.00005 and 0.0005 pg cell^−1^ (0.1 and 1 ng mL^−1^), respectively.

To compare DA cell quota and DA proportion of the strains between *Pseudo-nitzschia* species assessed above, the data sets were assessed by statistical analysis using BellCurve for Excel (Social Survey Research Information, Tokyo, Japan). Nonparametric analysis (Mann–Whitney U test) was performed on the data sets since they did not show a normal distribution.

### 4.4. Classification of Potential ASP Risk and Distribution Mapping of Each Pseudo-nitzschia Species in New Zealand Coastal Waters

The potential ASP risk for the 14 *Pseudo-nitzschia* species morphologically and/or genetically identified and two species morphologically identified previously was classified based on toxin production assessed in the present and previous studies in New Zealand, following the criteria reported by Rhodes et al. (2013) [10]. Briefly, species with a maximum DA production of ≥0.5 pg cell^−1^ was classified as potential ‘high ASP risk’ species. Species with a maximum DA production of ≤0.1 pg cell^−1^ was classified as potential ‘low ASP risk’ species. Species with DA production not detected or with trace levels of DA cell quota were classified as potential ‘no ASP risk’ species.

The distribution pattern of each species, having different potential ASP risks in New Zealand, was assessed and plotted on the map, based on the results of the present and previous studies.

## Figures and Tables

**Table 1:** Summary of domoic acid (DA) and DA isomers production determined for isolates of 16 *Pseudo-nitzschia* species from New Zealand coastal waters between 1993 and 2020 (analysed by LC-MS/MS and/or LC-UV). Double circle: historical locality of the isolates recorded by 2011. Black circle: locality of the isolates recorded in the present study. This table was updated from previous review papers (Table 3 in Rhodes et al. 2012; Table 1 in Rhodes et al. 2013).

Potential ASP Risk in New Zealand ^a^	Species	First Record in New Zealand	Range of DA and DA Isomers Cell Quota (pg cell^−1^) ^b, c^								Locality of Clonal Isolates in New Zealand ^d^											
			DA	epi-DA	iso-DA A	iso-DA B ^e^	iso-DA C	iso-DA D	iso-DA E		Subtropical				Warm Temperate						Cold Temperate	
											Northland ^f^	Hauraki Gulf	Bay of Plenty ^f^		Poverty Bay/Hawke Bay	Golden Bay ^f^/Tasman Bay ^f^	Marlborough Sounds ^f^	Canterbury	Caroline Bay		Otago	Big GloryBay ^f^
High	*P. australis*	1993	0.004–2.20(0–35.00) ^g^	0–0.010	0–0.060	trace	0–1.700 (9.050 ^h^)	0–0.003	0–trace		◎	◎	◎ ^i^ ●			●	◎ ●	◎ ^i^				◎ ●
	*P. multiseries*	1997	0.47–7.20	0–0.012	0.014–0.206	0–trace	0–0.239	0.003–0.020	trace–0.005		◎ ^i^					◎ ●	●					
	*P. multistriata*	1997	0–1.60	0	0–trace	0	0–0.200	0	0		◎ ●	◎	◎		◎ ^i^	◎ ●	◎ ●					
	*P. pungens*	1993	0–0.47	0	0	0	0	0	0		●		◎ ^i^		◎	◎ ●	◎ ●	◎				◎ ●
Low	*P. delicatissima* ^j^	1996	0–0.12	0	0	0	0	0	0		◎ ^i^ ●		◎			◎ ^i^	◎ ●				◎ ^i^	◎ ●
	*P. fraudulenta*	1996	0–0.03	0	0	0	0	0	0		◎ ●	◎ ^i^	◎ ^i^				◎ ●	◎				◎ ●
	‘*P. pseudodelicatissima*’ ^k^	1996	0.12	NT	NT	NT	0	NT	NT								◎		◎		◎ ^i^	◎
No	*P. americana*	1994	0	0	0	0	0	0	0		●	◎				●	◎ ●	◎				◎
	*P. arenysensis*	2019	0	0	0	0	0	0	0		●						●					
	‘*P. caciantha*’ ^k, l^	2005	0	NT	NT	NT	0	NT	NT								◎					
	*P. calliantha* ^l^	2005	0	0	0	0	0	0	0								◎ ●	◎	◎			
	*P. cuspidata* ^m^	2020 ^l^	0–trace	0	0	0	0	0	0								●					
	*P. galaxiae*	2020	0	0	0	0	0	0	0		●											
	*P. hasleana*	2020	0	0	0	0	0	0	0							●						●
	*P. plurisecta* ^m^	2005 ^l^	0	0	0	0	0	0	0		●						◎					
	*P.* (cf.) *subpacifica* ^n^	1996	0	0	0	0	0	0	0		◎ ●		●			●	◎ ●	◎ ^i^				◎ ^i^

^a^ Potential ‘high, low, and no ASP risk’ species represent potential high, low, and no toxin production species, respectively. ^b^ 0 indicates that no toxins were detected at the limit of detection (LoD). ^c^ NT indicates samples not tested. ^d^ The locality data include that of isolates both tested and not tested for toxins. ^e^ Iso-DA B was detected without quantitation due to lack of standard material. ^f^ Sampling site in the present study. ^g^ Rhodes et al. (1998b) reported a range of DA cell quota of wild *P. australis* cells with high concentration of up to 35.00 pg cell^−1^. ^h^ Rhodes et al. (2004) reported high concentration of iso-DA C cell quota of 9.050 pg cell^−1^ from a *P. australis* isolate cultured in copper addition f/2−Si medium. ^i^ Additional localities of the isolates were reported by previous studies (Rhodes et al. 1998a, 1998b, 2000, 2004, 2012, and/or Casteleyn et al. 2008). ^j^ ‘*Pseudo-nitzschia turgidula*’ reported previously in New Zealand is assigned as *P. delicatissima* subclade I in the present study based on an LSU rDNA D1–D2 sequence (DDBJ/EMBL/GenBank accession number: U92259), as discussed in Rhodes et al. (1998a, 1998b). ^k^ Species identification should be treated as tentative as discussed in Section 3.1. ^l^ Rhodes et al. (2013) reported *P. caciantha* strains by morphological identification from Marlborough Sounds, Canterbury, and Caroline Bay. Subsequently, the strains from the latter two sites were confirmed as *P. calliantha* based on sequences of the ITS region (Rhodes, unpublished data). Therefore, these ‘*P. caciantha*’ strains from the two sites are assigned as *P. calliantha* in the present study. ^m^ Rhodes et al. (2013) reported a *P. cuspidata* strain as *Pseudo-nitzschia* sp. strain Hobart-5 type by morphological identification. Subsequently, strain Hobart-5 was assigned as *P. plurisecta* by Orive et al. (2013). Therefore, *P. cuspidata* reported previously in New Zealand is assigned as *P. plurisecta* in the present study. ^n^ *Pseudo-nitzschia* (cf.) *heimii* reported previously in New Zealand is assigned as *P. subpacifica* in the present study based on morphological characters as reported in Rhodes et al. (2013).

## Data Availability

The data presented in this study are in the article and supplementary materials.

## Supplementary Materials

The following are available online at https://www.mdpi.com/article/10.3390/toxins13090637/s1, Table S1: Details of 22 sampling sites in New Zealand between 2018 and 2020. Table S2: Details of 130 clonal strains of *Pseudo-nitzschia* established from New Zealand between 2018 and 2020. Table S3: Average, SD, minimum, and maximum uncorrected *p* distance of the ITS region of selected combinations between *Pseudo-nitzschia* species/clades/subclades. Table S4: Average, SD, minimum, and maximum uncorrected *p* distance of the LSU rDNA D1–D3 of selected combinations between *Pseudo-nitzschia* *hasleana* and several related species. Table S5: Details of 73 clonal strains of *Pseudo-nitzschia* from New Zealand tested for domoic acid (DA) and DA isomers analysis in the present study.

## Abbreviations

AIC, Akaike information criterion; ASDSF, average standard deviation of split frequencies; ASP, amnesic shellfish poisoning; BI, Bayesian inference; CICCM, Cawthron Institute Culture Collection of Microalgae; CNC, Cawthron Natural Compounds; *cox1*, cytochrome *c* oxidase subunit 1; DA, domoic acid; DDBJ, DNA Data Bank of Japan; EMBL, European Molecular Biology Laboratory; epi-DA, c5′-epidomoic acid; FISH, Fluorescence In Situ Hybridization; iso-DAs, isodomoic acids; ITS region, internal transcribed spacer 1–5.8S rDNA-ITS 2 region; LC-MS/MS, liquid chromatography-tandem mass spectrometry; LLoQ, lower limit of quantitation; LoD, limit of detection; LSU rDNA D1–D3, large subunit rDNA D1–D3 region; MCMCMC, Metropolis-coupled Markov chain Monte Carlo; MeCN, acetonitrile; ML, maximum likelihood; MRM, multiple reaction monitoring; NRC, National Research Council of Canada; PDA, photo diode array; pp, posterior probability; *rbcL*, RuBisCO large subunit; *rbcS*, RuBisCO small subunit; rDNA, ribosomal RNA gene; RuBisCO, ribulose-1,5-bisphosphate carboxylase-oxygenase; SSU, small subunit; TEF, toxicity equivalency factors; UGD, uncorrected genetic distance.
