# Supplementary material for: Update of the Planktonic Diatom Genus Pseudo-nitzschia in Aotearoa New Zealand Coastal Waters: Genetic Diversity and Toxin Production
Source: Toxins (Basel). 2021 Sep 10;13(9):637. doi: 10.3390/toxins13090637 (PMC8473122; doi:10.3390/toxins13090637)
Supplement: Supplementary file 1 [file toxins-13-00637-s001.zip › toxins-1340895-supplementary.pdf]

Table S1. Details of 22 sampling sites in New Zealand between 2018 and 2020.

Climate zone	Island	Sampling site code[a]	Sampling date	Location of sampling site	Latitude, Longitude	Sampling depth (m)
Subtropical	North Island	1	2018 or 2019, and 2020 January 14	Kerikeri, Far North, Northland	-35.1986, 174.0602	0−9 (0+3+6+9)
		2	2018 November 27 and 2020 June 22	Tapeka Point, Russell, Far North, Northland	-35.2451, 174.1179	0
		3	2020 July 20	Bream Bay, Whangārei, Northland	-35.8838, 174.4697	7
		4	2020 July 13	Ōpōtiki, Ōpōtiki, Bay of Plenty	-37.9079, 177.2161	0, 5, 10, and 15
Warm temperate	South Island	5	2018 or 2019, and 2020 June 21	Collingwood Farms, Tasman, Tasman	-40.6550, 172.7172	0−12
		6	2020 June 21	Tākaka, Tasman, Tasman	-40.8162, 172.8126	0−12
		7	2020 June 21	Bomber Port, Motueka, Tasman, Tasman	-41.1193, 173.0447	0−12
		8	2020 June 23 and 30	Oyster Bay, Marlborough, Marlborough	-41.0458, 173.7196	0−15
		9	2020 June 30	Hallam Cove, Marlborough, Marlborough	-40.9958, 173.8246	0−15
		10	2019 April 16 and 2020 July 20	Pukatea Bay, Tasman, Tasman	-40.9564, 173.8967	0−15
		11	2020 June 23	Cannon Hill, Marlborough, Marlborough	-40.9475, 173.9738	0−15
		12	2020 July 07	Brightlands Bay, Marlborough, Marlborough	-41.0612, 173.8544	0−15
		13	2020 January	Richmond Bay, Marlborough, Marlborough	-41.0102, 173.9665	0−15
		14	2020 January and 2020 July 13	Crail Bay, Marlborough, Marlborough	-41.1147, 173.9656	0−15
		15	2019 October and 2020 July 13	Beatrix Bay, Marlborough, Marlborough	-41.0306, 174.0054	0−15
		16	2020 July 13	Laverique Bay, Marlborough, Marlborough	-41.0530, 174.0455	0−15
		17	2019 October	Waitaria Bay, Marlborough, Marlborough	-41.1677, 174.0330	0−15
		18	2020 June 24	Horahora, Marlborough, Marlborough	-41.3307, 174.1360	0−15
		19	2019 May 01	Opihi Bay, Marlborough, Marlborough	-41.2938, 174.1452	0−15
		20	2019 May 01 and 2020 June 24	Whangakoko Bay, Marlborough, Marlborough	-41.2961, 174.1656	0−15
Cold temperate	Stewart Island	21	2020 July 06, 19, and November 22	Big Glory Bay, Southland, Southland	-46.9700, 168.1172	0−10
		22	2019 March 17 and 2020 November 22	Big Glory Bay, Southland, Southland	-46.98, 168.11	0−10

[a] Sampling site code shown in Figure 4.

Table S2. Details of 130 clonal strains of *Pseudo-nitzschia* established from New Zealand between 2018 and 2020. NR: sequence determined but not registered as it was identical to the other registered sequences of each species/clade/subclade. ND: not detected. -: sequence not determined or toxin production not tested.

Species	Clade/ subclade	Sequence type (ST)[a] of the ITS region	Strain name in CICCM[b]	Strain name	Sampling site code[c]	Sampling date	Isolater	DDBJ[d] accession number for the ITS region	DDBJ[d] accession number for the LSU rDNA D1–D3	DA and/or DA isomers production[e]
P. americana				PB024Ps03	3	2020 July 20	T. Nishimura	LC636495	-	ND
P. americana				PB024Ps04	3	2020 July 20	T. Nishimura	NR	-	-
P. americana				PB024Ps09	3	2020 July 20	T. Nishimura	NR	-	-
P. americana				G005Ps02	7	2020 June 21	T. Nishimura	LC636496	-	ND
P. americana			CAWB138	G018Ps01	10	2020 July 20	T. Nishimura	LC636497	LC636556	ND
P. americana				G031Ps01	16	2020 July 13	H. Bowers and T. Nishimura	NR	-	ND
P. arenysensis			CAWB139	PA030Ps01	2	2020 June 22	T. Nishimura	LC636498	-	ND
P. arenysensis				PA030Ps04	2	2020 June 22	T. Nishimura	LC636499	-	ND
P. arenysensis				PA030Ps05	2	2020 June 22	T. Nishimura	NR	-	-
P. arenysensis			CAWB131	Psd-5	10	2019 April 16	M. Balci	LC636500	LC636557	ND
P. arenysensis			CAWB132	Psd-6	10	2019 April 16	M. Balci	LC636501	LC636558	ND
P. arenysensis			CAWB133	Psd-7	10	2019 April 16	M. Balci	-	LC636559	ND
P. arenysensis			CAWB134	Psd-8	10	2019 April 16	M. Balci	-	LC636560	ND
P. australis		ST1		PD009Ps05	4	2020 July 13	T. Nishimura	LC636502	-	Produced
P. australis		ST1	CAWB140	G007Ps03	6	2020 June 21	T. Nishimura	LC636503	LC636561	Produced
P. australis		ST1		G010Ps02	9	2020 June 30	T. Nishimura	NR	-	-
P. australis		ST1		G010Ps03	9	2020 June 30	T. Nishimura	NR	-	-
P. australis		ST1		G027Ps01	12	2020 July 07	T. Nishimura	LC636504	-	Produced
P. australis		ST1		G027Ps02	12	2020 July 07	T. Nishimura	NR	-	-
P. australis		ST1		G015Ps04	14	2020 July 13	H. Bowers and T. Nishimura	LC636505	LC636562	Produced
P. australis		ST1		G037Ps01	16	2020 July 13	H. Bowers and T. Nishimura	LC636506	-	Produced
P. australis		ST1		G037Ps02	16	2020 July 13	H. Bowers and T. Nishimura	NR	-	-
P. australis		ST1		J328Ps20	21	2020 November 22	T. Nishimura	LC636507	-	Produced
P. australis		ST1		J237Ps07	22	2020 November 22	T. Nishimura	LC636508	-	Produced
P. australis		ST2		G018Ps02	10	2020 July 20	T. Nishimura	LC636509	LC636563	Produced
P. australis		ST2		G018Ps03	10	2020 July 20	T. Nishimura	NR	-	-
P. australis		ST2		G018Ps04	10	2020 July 20	T. Nishimura	LC636510	-	Produced
P. calliantha		ST1	CAWB135	Crail Bay	14	2020 January	L. L. Rhodes	LC636511	LC636564	ND
P. calliantha		ST2		Waitaria Bay	17	2019 October	S. Challenger	LC636512	LC636565	ND
P. calliantha		ST3		Richmond Bay	13	2020 January	L. L. Rhodes	LC636513	LC636566	ND
P. cuspidata	clade Ia		CAWB141	PA030Ps10	2	2020 June 22	T. Nishimura	LC636514	LC636567	Produced (trace)

P. delicatissima	subclade I	CAWB136	PA014Ps04	1	2020 January 14	T. Nishimura	LC636515	LC636568	ND
P. delicatissima	subclade I		PA014Ps05	1	2020 January 14	T. Nishimura	LC636516	LC636569	ND
P. delicatissima	subclade I		PA014Ps11	1	2020 January 14	T. Nishimura	LC636517	LC636570	ND
P. delicatissima	subclade I	CAWB142	PA030Ps06	2	2020 June 22	T. Nishimura	LC636518	-	ND
P. delicatissima	subclade I		PA030Ps11	2	2020 June 22	T. Nishimura	LC636519	-	ND
P. delicatissima	subclade II	CAWB143	G013Ps03	8	2020 June 23	T. Nishimura	LC636520	LC636571	ND
P. delicatissima	subclade II		G013Ps04	8	2020 June 23	T. Nishimura	LC636521	-	ND
P. delicatissima	subclade II		G013Ps05	8	2020 June 23	T. Nishimura	NR	-	-
P. delicatissima	subclade II		G013Ps08	8	2020 June 23	T. Nishimura	NR	-	-
P. delicatissima	subclade II		G013Ps10	8	2020 June 23	T. Nishimura	NR	-	-
P. delicatissima	subclade II		G013Ps12	8	2020 June 23	T. Nishimura	NR	-	-
P. delicatissima	subclade II		G013Ps13	8	2020 June 30	H. Bowers and T. Nishimura	NR	-	-
P. delicatissima	subclade II		G013Ps14	8	2020 June 30	H. Bowers and T. Nishimura	NR	-	-
P. delicatissima	subclade II		G013Ps15	8	2020 June 30	H. Bowers and T. Nishimura	NR	-	-
P. delicatissima	subclade II		G013Ps16	8	2020 June 30	T. Nishimura	NR	-	-
P. delicatissima	subclade II	CAWB144	J328Ps01	21	2020 July 06	H. Bowers and T. Nishimura	LC636522	LC636572	ND
P. delicatissima	subclade II		J328Ps02	21	2020 July 06	H. Bowers and T. Nishimura	LC636523	-	ND
P. delicatissima	subclade II		J328Ps03	21	2020 July 06	H. Bowers and T. Nishimura	NR	-	-
P. delicatissima	subclade II		J328Ps04	21	2020 July 06	H. Bowers and T. Nishimura	NR	-	-
P. delicatissima	subclade II		J328Ps05	21	2020 July 06	H. Bowers and T. Nishimura	NR	-	-
P. delicatissima	subclade II		J328Ps06	21	2020 July 06	H. Bowers and T. Nishimura	NR	-	-
P. delicatissima	subclade II		J328Ps07	21	2020 July 19	T. Nishimura	NR	-	-
P. delicatissima	subclade II		J328Ps08	21	2020 July 19	T. Nishimura	NR	-	-
P. delicatissima	subclade II		J328Ps09	21	2020 July 19	T. Nishimura	NR	-	-
P. delicatissima	subclade II		J328Ps10	21	2020 July 19	T. Nishimura	NR	-	-
P. delicatissima	subclade II		J328Ps11	21	2020 July 19	T. Nishimura	NR	-	-
P. delicatissima	subclade II		J328Ps14	21	2020 November 22	T. Nishimura	NR	-	-
P. delicatissima	subclade II		J328Ps17	21	2020 November 22	T. Nishimura	NR	-	-
P. fraudulenta			828	1	2018 or 2019	S. Challenger	-	LC636573	-
P. fraudulenta			G013Ps02	8	2020 June 23	T. Nishimura	NR	-	ND
P. fraudulenta			G013Ps09	8	2020 June 23	T. Nishimura	NR	-	-
P. fraudulenta		CAWB145	G015Ps01	14	2020 July 13	H. Bowers	LC636524	LC636574	ND
P. fraudulenta			G015Ps03	14	2020 July 13	H. Bowers	NR	-	-
P. fraudulenta			G011Ps04	20	2020 June 24	T. Nishimura	NR	-	ND
P. fraudulenta			J328Ps12	21	2020 July 19	T. Nishimura	NR	-	-
P. fraudulenta			J328Ps13	21	2020 July 19	T. Nishimura	NR	-	-
P. fraudulenta			J328Ps16	21	2020 November 22	T. Nishimura	LC636525	-	ND

P. fraudulenta				J328Ps18	21	2020 November 22	T. Nishimura	NR	-	-
P. fraudulenta				J328Ps19	21	2020 November 22	T. Nishimura	NR	-	-
P. fraudulenta			CAWB128	Kiwa1	22	2019 March 17	S. Challenger	-	LC636575	ND
P. fraudulenta				Kiwa2	22	2019 March 17	S. Challenger	-	LC636576	-
P. galaxiae			CAWB153	PA014Ps01	1	2020 January 14	T. Nishimura	LC636526	LC636577	ND
P. galaxiae			CAWB154	PA014Ps07	1	2020 January 14	T. Nishimura	LC636527	LC636578	ND
P. hasleana	clade I		CAWB146	G007Ps01	6	2020 June 21	T. Nishimura	LC636528	LC636579	ND
P. hasleana	clade I		CAWB147	G007Ps02	6	2020 June 21	T. Nishimura	LC636529	-	ND
P. hasleana	clade II		CAWB156	J328Ps15	21	2020 November 22	T. Nishimura	LC636530	LC636580	ND
P. hasleana	clade II			J237Ps01	22	2020 November 22	T. Nishimura	LC636531	-	-
P. hasleana	clade II			J237Ps02	22	2020 November 22	T. Nishimura	LC636532	-	-
P. hasleana	clade II		CAWB155	J237Ps05	22	2020 November 22	T. Nishimura	LC636533	LC636581	ND
P. multiseries			CAWB149	G001Ps03	5	2020 June 21	T. Nishimura	LC636534	LC636582	Produced
P. multiseries				G001Ps04	5	2020 June 21	T. Nishimura	LC636535	-	Produced
P. multiseries				G007Ps04	6	2020 June 21	T. Nishimura	LC636536	-	Produced
P. multiseries				G010Ps10	9	2020 June 30	T. Nishimura	LC636537	-	Produced
P. multistriata		ST1	CAWB137	PA014Ps09	1	2020 January 14	T. Nishimura	LC636538	LC636583	ND
P. multistriata		ST1		PA014Ps10	1	2020 January 14	T. Nishimura	LC636539	LC636584	ND
P. multistriata		ST1		G013Ps21	8	2020 June 30	T. Nishimura	LC636540	-	ND
P. multistriata		ST1		G013Ps22	8	2020 June 30	T. Nishimura	NR	-	-
P. multistriata		ST1		G027Ps06	12	2020 July 07	T. Nishimura	LC636541	-	Produced (trace)
P. multistriata		ST2	CAWB127	827	2	2018 November 27	S. Challenger	LC636542	LC636585	ND
P. multistriata		ST2	CAWB150	PB024Ps01	3	2020 July 20	T. Nishimura	LC636543	-	ND
P. multistriata		ST2		PB024Ps02	3	2020 July 20	T. Nishimura	NR	-	-
P. multistriata		ST2		PB024Ps05	3	2020 July 20	T. Nishimura	NR	-	-
P. multistriata		ST2		PB024Ps07	3	2020 July 20	T. Nishimura	NR	-	-
P. multistriata		ST2		PB024Ps08	3	2020 July 20	T. Nishimura	NR	-	-
P. multistriata		ST2		G001Ps01	5	2020 June 21	T. Nishimura	LC636544	-	Produced (trace)
P. multistriata		ST2		G001Ps02	5	2020 June 21	T. Nishimura	LC636545	LC636586	ND
P. plurisecta			CAWB151	G027Ps03	12	2020 July 07	T. Nishimura	LC636546	LC636587	ND
P. pungens	clade I	ST1	CAWB152	PA030Ps03	2	2020 June 22	T. Nishimura	LC636547	LC636588	ND
P. pungens	clade I	ST1		PA030Ps08	2	2020 June 22	T. Nishimura	NR	-	-
P. pungens	clade I	ST1		G007Ps06	6	2020 June 21	T. Nishimura	NR	-	ND
P. pungens	clade I	ST1		G005Ps01	7	2020 June 21	T. Nishimura	NR	-	ND
P. pungens	clade I	ST1		G005Ps04	7	2020 June 21	T. Nishimura	NR	-	-
P. pungens	clade I	ST1		G010Ps01	9	2020 June 30	T. Nishimura	NR	-	ND
P. pungens	clade I	ST1		G010Ps04	9	2020 June 30	T. Nishimura	NR	-	-

P. pungens	clade I	ST1		G010Ps06	9	2020 June 30	T. Nishimura	NR	-	-
P. pungens	clade I	ST1		G010Ps07	9	2020 June 30	T. Nishimura	NR	-	-
P. pungens	clade I	ST1		G010Ps08	9	2020 June 30	T. Nishimura	NR	-	-
P. pungens	clade I	ST1		G018Ps05	10	2020 July 20	T. Nishimura	NR	-	ND
P. pungens	clade I	ST1		Beatrix Bay	15	2019 October	S. Challenger	LC636548	-	ND
P. pungens	clade I	ST1		G011Ps01	20	2020 June 24	T. Nishimura	NR	-	ND
P. pungens	clade I	ST1		G011Ps02	20	2020 June 24	T. Nishimura	NR	-	-
P. pungens	clade I	ST1		G011Ps05	20	2020 June 24	T. Nishimura	NR	-	-
P. pungens	clade I	ST1		G011Ps07	20	2020 June 24	T. Nishimura	NR	-	-
P. pungens	clade I	ST1		G011Ps08	20	2020 June 24	T. Nishimura	NR	-	-
P. pungens	clade I	ST1		G011Ps09	20	2020 June 24	T. Nishimura	NR	-	-
P. pungens	clade I	ST1		J328Ps21	21	2020 November 22	T. Nishimura	LC636549	-	ND
P. pungens	clade I	ST1		J237Ps03	22	2020 November 22	T. Nishimura	NR	-	ND
P. pungens	clade I	ST1		J237Ps04	22	2020 November 22	T. Nishimura	NR	-	-
P. pungens	clade I	ST1		J237Ps06	22	2020 November 22	T. Nishimura	NR	-	-
P. pungens	clade I	ST1		J237Ps08	22	2020 November 22	T. Nishimura	NR	-	-
P. pungens	clade I	ST2		G027Ps05	12	2020 July 07	T. Nishimura	LC636550	-	ND
P. pungens	clade I	ST2		G015Ps02	14	2020 July 13	H. Bowers	LC636551	-	ND
P. pungens	clade I	-	CAWB130	Psd-4	19	2019 May 01	M. Balci	-	LC636589	ND
P. pungens	clade I	-	CAWB129	Psd-3	20	2019 May 01	M. Balci	-	LC636590	ND
P. cf. *subpacifica*				PA030Ps02	2	2020 June 22	T. Nishimura	LC636552	-	ND
P. cf. *subpacifica*				PA030Ps07	2	2020 June 22	T. Nishimura	LC636553	LC636591	ND
P. cf. *subpacifica*			CAWB148	PD009Ps04	4	2020 July 13	T. Nishimura	LC636554	LC636592	ND
P. cf. *subpacifica*				826	5	2018 or 2019	S. Challenger	-	LC636593	-
P. cf. *subpacifica*				G011Ps03	20	2020 June 24	T. Nishimura	LC636555	-	ND

[a] Sequences that were not identical but were not a clade/subclade level divergence were assigned as sequence type (ST).

[b] CICCM: the Cawthron Institute Culture Collection of Microalgae.

[c] Sampling site code shown in Figure 4.

[d] DDBJ: the DNA Data Bank of Japan.

[e] Details of the DA and DA isomers analysis are shown in Table S5.

Table S3. Average, SD, minimum, and maximum uncorrected *p* distance of the ITS region of selected combinations between *Pseudo-nitzschia* species/clades/subclades. NA: data not available.

Species/clade/subclade	Species/clade/subclade	Number of positions[a]	Number of combinations	*p* distance			
				Average	SD	Minimum	Maximum
P. arenysensis clade I	*P. arenysensis* clade I	690	36	0	0	0	0
	P. arenysensis clade II	690	18	0.045	0.005	0.040	0.052
	P. cf. *arenysensis*	690	27	0.040	0.000	0.040	0.040
P. arenysensis clade II	*P. arenysensis* clade II	690	1	NA	NA	NA	0.013
	P. cf. *arenysensis*	690	6	0.041	0.000	0.040	0.041
P. cf. *arenysensis*	*P.* cf. *arenysensis*	690	3	0	0	0	0
P. cuspidata clade I[b]	*P. cuspidata* clade I[b]	724	21	0.009	0.006	0	0.021
	P. cuspidata clade II	724	35	0.030	0.004	0.024	0.037
	P. pseudodelicatissima [c]	724	63	0.031	0.003	0.026	0.037
	P. plurisecta	724	35	0.031	0.002	0.026	0.034
P. cuspidata clade Ia	*P. cuspidata* clade Ia	724	3	0.001	0.001	0	0.001
	P. cuspidata clade Ib	724	12	0.013	0.004	0.010	0.021
P. cuspidata clade Ib	*P. cuspidata* clade Ib	724	6	0.006	0.003	0	0.010
P. cuspidata clade II	*P. cuspidata* clade II	724	10	0.012	0.008	0.003	0.024
	P. pseudodelicatissima [c]	724	45	0.021	0.005	0.015	0.031
	P. plurisecta	724	25	0.036	0.003	0.030	0.041
P. pseudodelicatissima [c]	*P. pseudodelicatissima* [c]	724	36	0.004	0.003	0	0.009
	P. plurisecta	724	45	0.034	0.004	0.027	0.039
P. plurisecta	*P. plurisecta*	724	10	0.004	0.002	0	0.007
P. decipiens clade A	*P. decipiens* clade A	644	1	NA	NA	NA	0.005
	P. decipiens clade B	644	4	0.027	0.002	0.023	0.030
P. decipiens clade B	*P. decipiens* clade B	644	1	NA	NA	NA	0.014
P. delicatissima subclade I	*P. delicatissima* subclade I	671	45	0.002	0.002	0	0.005
	P. delicatissima subclade II	671	80	0.042	0.001	0.041	0.046
	P. delicatissima subclade III	671	60	0.044	0.001	0.043	0.046
P. delicatissima subclade II	*P. delicatissima* subclade II	671	28	0.001	0.001	0	0.003
	P. delicatissima subclade III	671	48	0.033	0.001	0.031	0.036
P. delicatissima subclade III	*P. delicatissima* subclade III	671	15	0.001	0.001	0	0.003
Four New Zealand strains of *P. delicatissima* subclade II	Four other strains of *P. delicatissima* subclade II	671	16	0.002	0.001	0.002	0.003
P. galaxiae clade A[d]	*P. galaxiae* clade A[d]	593	1	NA	NA	NA	0.012
	P. galaxiae clade B[d]	593	12	0.047	0.002	0.045	0.050
	P. galaxiae clade C	593	6	0.068	0.004	0.064	0.072
P. galaxiae clade B[d]	*P. galaxiae* clade B[d]	593	15	0.010	0.009	0	0.024
	P. galaxiae clade C	593	18	0.056	0.005	0.053	0.067
P. galaxiae clade C	*P. galaxiae* clade C	593	3	0	0	0	0
P. hasleana clade I	*P. hasleana* clade I	620	36	0.002	0.001	0	0.005
	P. hasleana clade II	620	36	0.012	0.001	0.011	0.015
P. hasleana clade II	*P. hasleana* clade II	620	6	0	0	0	0
P. pungens clade I	*P. pungens* clade I	624	28	0.001	0.001	0	0.003
	P. pungens clade II	624	24	0.012	0.001	0.011	0.014
	P. pungens clade III[e]	624	72	0.033	0.005	0.026	0.047
P. pungens clade II	*P. pungens* clade II	624	3	0.001	0.001	0	0.002
	P. pungens clade III[e]	624	27	0.035	0.005	0.027	0.048
P. pungens clade III[e]	*P. pungens* clade III[e]	624	36	0.014	0.006	0	0.027
P. pungens clade IIIaa	*P. pungens* clade IIIaa	624	3	0.006	0.001	0.005	0.008
	P. pungens clade IIIab	624	12	0.015	0.005	0.011	0.024
	P. pungens clade IIIb	624	6	0.013	0.001	0.011	0.014
P. pungens clade IIIab	*P. pungens* clade IIIab	624	6	0.010	0.005	0.005	0.016
	P. pungens clade IIIb	624	8	0.019	0.005	0.016	0.027
P. pungens clade IIIb	*P. pungens* clade IIIb	624	1	NA	NA	NA	0
P. simulans subclade I	*P. simulans* subclade I	703	1	NA	NA	NA	0
	P. simulans subclade II	703	4	0.039	0.000	0.039	0.039
P. simulans subclade II	*P. simulans* subclade II	703	1	NA	NA	NA	0.001

[a] Number of positions used for the final data set for the calculation of uncorrected *p* distances.

[b] *Pseudo-nitzschia cuspidata* clade I contains clades Ia and Ib. Clade Ib includes two *P. cuspidata* var. *manzanillensis* strains (Ps116 and Ps142) and two *P. cuspidata* strains (MC3041 and MC3207).

[c] *Pseudo-nitzschia pseudodelicatissima* includes two *P. cuspidata* strains (Tenerife8 and NWFSC194).

[d] *Pseudo-nitzschia galaxiae* clades A and B correspond to clades I and II reported by McDonald et al. (2007), respectively.

[e] *Pseudo-nitzschia pungens* clade III contains clades IIIaa, IIIab, and IIIb.

Table S4. Average, SD, minimum, and maximum uncorrected *p* distance of the LSU rDNA D1−D3 of selected combinations between *Pseudo-nitzschia hasleana* and several related species. NA: data not available.

Species/subclade	Species/subclade	Number of positions[a]	Number of combinations	*p* distance			
				Average	SD	Minimum	Maximum
P. hasleana clade I	*P. hasleana* clade I	659	10	0	0	0	0
	P. hasleana clade II	659	10	0.006	0	0.006	0.006
	'*P. calliantha*'[b]	659	5	0.014	< 0.001	0.014	0.014
	P. calliantha	659	25	0.018	0.001	0.015	0.020
	P. mannii	659	10	0.025	0.001	0.024	0.026
	P. limii	659	10	0.007	0.001	0.006	0.008
	P. kodamae	659	10	0.018	0	0.018	0.018
P. hasleana clade II	*P. hasleana* clade II	659	1	NA	NA	NA	0
	'*P. calliantha*'[b]	659	2	0.008	0	0.008	0.008
	P. calliantha	659	10	0.019	0.001	0.017	0.020
	P. mannii	659	4	0.027	0.001	0.026	0.027
	P. limii	659	4	0.008	0.001	0.008	0.009
	P. kodamae	659	4	0.021	0	0.021	0.021
'*P. calliantha*'[b]	'*P. calliantha*'[b]	659	NA	NA	NA	NA	NA
	P. calliantha	659	5	0.027	0.001	0.025	0.028
	P. mannii	659	2	0.035	0.001	0.034	0.035
	P. limii	659	2	0.016	0.001	0.015	0.017
	P. kodamae	659	2	0.027	< 0.001	0.027	0.027
P. calliantha	*P. calliantha*	659	10	0.001	0.001	0	0.003
	P. mannii	659	10	0.016	0.001	0.014	0.018
	P. limii	659	10	0.014	0.002	0.011	0.017
	P. kodamae	659	10	0.027	0.001	0.024	0.029
P. mannii	*P. mannii*	659	1	NA	NA	NA	0.002
	P. limii	659	4	0.018	0.001	0.017	0.020
	P. kodamae	659	4	0.014	0.001	0.014	0.015
P. limii	*P. limii*	659	1	NA	NA	NA	0.002
	P. kodamae	659	4	0.014	0.001	0.014	0.015
P. kodamae	*P. kodamae*	659	1	NA	NA	NA	0.003

[a] Number of positions used for the final data set for the calculation of uncorrected *p* distances.

[b] '*Pseudo-nitzschia calliantha*' indicates strain CAWB114 from New Zealand (Rhodes et al. 2013).

Table S5. Details of 73 clonal strains of *Pseudo-nitzschia* from New Zealand tested for domoic acid (DA) and DA isomers analysis in the present study. 0: no toxins were detected at the limit of detection (LoD). trace: toxin cell quota was detected between LoD and lower limit of quantitation (LLoQ). NA: data not available. NC: data not calculated. Bold letter: detected or quantified value.

Species	Clade/ subclade	Sequence type (ST)[a] of the ITS	Strain name in CICCM[b]	Strain name	Medium	Harvested cell number	Day	Number of months after isolation[c]	LoD	LLoQ	Cell quota (pg cell^{-1})							Proportion (%)						
											DA	epi-DA	iso-DA A	iso-DA B	iso-DA C	iso-DA D	iso-DA E	DA	epi-DA	iso-DA A	iso-DA B	iso-DA C	iso-DA D	iso-DA E
P. americana				PB024Ps03	f/2−Si	9,653,333	9	3	0.00005	0.0005	0	0	0	0	0	0	0	NA	NA	NA	NA	NA	NA	NA
P. americana				G005Ps02	f/2−Si	7,056,000	10	8	0.00005	0.0005	0	0	0	0	0	0	0	NA	NA	NA	NA	NA	NA	NA
P. americana			CAWB138	G018Ps01	f/2−Si	8,576,000	9	3	0.00005	0.0005	0	0	0	0	0	0	0	NA	NA	NA	NA	NA	NA	NA
P. americana				G031Ps01	f/2−Si	8,224,000	10	7	0.00005	0.0005	0	0	0	0	0	0	0	NA	NA	NA	NA	NA	NA	NA
P. arenysensis	clade I		CAWB139	PA030Ps01	f/2−Si	13,813,333	9	4	0.00005	0.0005	0	0	0	0	0	0	0	NA	NA	NA	NA	NA	NA	NA
					f/2−Si	2,288,000	43	5	0.00005	0.0005	0	0	0	0	0	0	0	NA	NA	NA	NA	NA	NA	NA
P. arenysensis	clade I			PA030Ps04	f/2−Si	9,226,667	9	4	0.00005	0.0005	0	0	0	0	0	0	0	NA	NA	NA	NA	NA	NA	NA
					f/2−Si	1,981,333	43	5	0.00005	0.0005	0	0	0	0	0	0	0	NA	NA	NA	NA	NA	NA	NA
P. arenysensis	clade I		CAWB131	Psd-5	f/2	16,350,000	10	14	0.0005	0.0025	0	0	0	0	0	0	0	NA	NA	NA	NA	NA	NA	NA
P. arenysensis	clade I		CAWB132	Psd-6	f/2	37,050,000	10	14	0.0005	0.0025	0	0	0	0	0	0	0	NA	NA	NA	NA	NA	NA	NA
					f/2−Si	18,144,000	10	22	0.00005	0.0005	0	0	0	0	0	0	0	NA	NA	NA	NA	NA	NA	NA
P. arenysensis	clade I		CAWB133	Psd-7	f/2	55,500,000	10	14	0.0005	0.0025	0	0	0	0	0	0	0	NA	NA	NA	NA	NA	NA	NA
P. arenysensis	clade I		CAWB134	Psd-8	f/2	20,550,000	10	14	0.0005	0.0025	0	0	0	0	0	0	0	NA	NA	NA	NA	NA	NA	NA
P. australis		ST1		PD009Ps05	f/2−Si	1,260,000	9	3	0.00008	0.0008	**0.27**	0	**0.009**	**trace**	**0.119**	0	0	**68.1**	NA	**2.3**	**NC**	**29.7**	NA	NA
P. australis		ST1	CAWB140	G007Ps03	f/2−Si	420,000	9	4	0.0002	0.002	**0.06**	0	**0.003**	**trace**	**0.021**	0	0	**72.6**	NA	**3.0**	**NC**	**24.4**	NA	NA
P. australis		ST1		G027Ps01	f/2−Si	573,333	9	3	0.0002	0.002	**0.73**	0	**0.024**	**trace**	**0.884**	0	0	**44.4**	NA	**1.5**	**NC**	**54.1**	NA	NA
P. australis		ST1		G015Ps04	f/2−Si	304,000	9	3	0.0003	0.003	**0.05**	0	0	**trace**	**0.010**	0	0	**83.2**	NA	NA	**NC**	**16.8**	NA	NA
					f/2−Si	1,506,667	10	7	0.00007	0.0007	**0.004**	0	**trace**	**trace**	**0.002**	0	0	**67.1**	NA	**NC**	**NC**	**32.9**	NA	NA
P. australis		ST1		G037Ps01	f/2−Si	966,667	9	3	0.0001	0.001	**0.09**	0	**0.003**	**trace**	**0.051**	0	0	**62.1**	NA	**2.2**	**NC**	**35.8**	NA	NA
P. australis		ST1		J328Ps20	f/2−Si	580,000	10	3	0.0002	0.002	**0.14**	**trace**	**0.006**	**trace**	**0.007**	**trace**	**trace**	**91.5**	**NC**	**3.7**	**NC**	**4.8**	**NC**	**NC**
P. australis		ST1		J237Ps07	f/2−Si	372,000	10	3	0.0003	0.003	**2.02**	**0.010**	**0.060**	**trace**	**0.118**	**0.003**	**trace**	**91.3**	**0.4**	**2.7**	**NC**	**5.4**	**0.1**	**NC**
P. australis		ST2		G018Ps02	f/2−Si	1,173,333	9	3	0.00009	0.0009	**0.04**	0	**0.001**	**trace**	**0.006**	0	0	**83.8**	NA	**2.7**	**NC**	**13.5**	NA	NA
P. australis		ST2		G018Ps04	f/2−Si	392,000	10	7	0.0003	0.003	**0.11**	0	**0.003**	**trace**	**0.050**	**trace**	0	**67.9**	NA	**2.0**	**NC**	**30.1**	**NC**	NA
P. calliantha		ST1	CAWB135	Crail Bay	f/2	41,400,000	10	5	0.0005	0.0025	0	0	0	0	0	0	0	NA	NA	NA	NA	NA	NA	NA
					f/2−Si	6,240,000	9	9	0.00005	0.0005	0	0	0	0	0	0	0	NA	NA	NA	NA	NA	NA	NA
P. calliantha		ST2		Waitaria Bay	f/2	39,300,000	10	8	0.0005	0.0025	0	0	0	0	0	0	0	NA	NA	NA	NA	NA	NA	NA
					f/2−Si	11,386,667	9	12	0.00005	0.0005	0	0	0	0	0	0	0	NA	NA	NA	NA	NA	NA	NA
P. calliantha		ST3		Richmond Bay	f/2	40,800,000	10	5	0.0005	0.0025	0	0	0	0	0	0	0	NA	NA	NA	NA	NA	NA	NA
					f/2−Si	13,216,000	10	13	0.00005	0.0005	0	0	0	0	0	0	0	NA	NA	NA	NA	NA	NA	NA
P. cuspidata	clade Ia		CAWB141	PA030Ps10	f/2−Si	12,026,667	9	4	0.00005	0.0005	0	0	0	0	0	0	0	NA	NA	NA	NA	NA	NA	NA
					f/2−Si	1,317,333	43	5	0.00008	0.0008	**trace**	0	0	0	0	0	0	**NC**	NA	NA	NA	NA	NA	NA
P. delicatissima	subclade I		CAWB136	PA014Ps04	f/2	120,900,000	10	5	0.0005	0.0025	0	0	0	0	0	0	0	NA	NA	NA	NA	NA	NA	NA
					f/2−Si	29,333,333	9	9	0.00005	0.0005	0	0	0	0	0	0	0	NA	NA	NA	NA	NA	NA	NA
P. delicatissima	subclade I			PA014Ps05	f/2	52,125,000	10	5	0.0005	0.0025	0	0	0	0	0	0	0	NA	NA	NA	NA	NA	NA	NA
					f/2−Si	4,213,333	9	9	0.00005	0.0005	0	0	0	0	0	0	0	NA	NA	NA	NA	NA	NA	NA
P. delicatissima	subclade I			PA014Ps11	f/2	102,000,000	10	5	0.0005	0.0025	0	0	0	0	0	0	0	NA	NA	NA	NA	NA	NA	NA
P. delicatissima	subclade I		CAWB142	PA030Ps06	f/2−Si	5,093,333	9	4	0.00005	0.0005	0	0	0	0	0	0	0	NA	NA	NA	NA	NA	NA	NA
P. delicatissima	subclade I			PA030Ps11	f/2−Si	13,066,667	9	4	0.00005	0.0005	0	0	0	0	0	0	0	NA	NA	NA	NA	NA	NA	NA
P. delicatissima	subclade II		CAWB143	G013Ps03	f/2−Si	12,880,000	9	4	0.00005	0.0005	0	0	0	0	0	0	0	NA	NA	NA	NA	NA	NA	NA
					f/2−Si	853,333	43	5	0.0001	0.001	0	0	0	0	0	0	0	NA	NA	NA	NA	NA	NA	NA
P. delicatissima	subclade II			G013Ps04	f/2−Si	17,386,667	9	4	0.00005	0.0005	0	0	0	0	0	0	0	NA	NA	NA	NA	NA	NA	NA
					f/2−Si	829,333	43	5	0.0001	0.001	0	0	0	0	0	0	0	NA	NA	NA	NA	NA	NA	NA
P. delicatissima	subclade II		CAWB144	J328Ps01	f/2−Si	10,666,667	9	3	0.00005	0.0005	0	0	0	0	0	0	0	NA	NA	NA	NA	NA	NA	NA
					f/2−Si	3,280,000	43	4	0.00005	0.0005	0	0	0	0	0	0	0	NA	NA	NA	NA	NA	NA	NA

P. delicatissima	subclade II			J328Ps02	f/2−Si	15,680,000	9	3	0.00005	0.0005	0	0	0	0	0	0	0	NA	NA	NA	NA	NA	NA	NA
					f/2−Si	3,440,000	43	4	0.00005	0.0005	0	0	0	0	0	0	0	NA	NA	NA	NA	NA	NA	NA
P. fraudulenta				G013Ps02	f/2−Si	1,328,000	10	8	0.00008	0.0008	0	0	0	0	0	0	0	NA	NA	NA	NA	NA	NA	NA
P. fraudulenta			CAWB145	G015Ps01	f/2−Si	1,306,667	9	3	0.00008	0.0008	0	0	0	0	0	0	0	NA	NA	NA	NA	NA	NA	NA
P. fraudulenta				G011Ps04	f/2−Si	4,640,000	9	4	0.00005	0.0005	0	0	0	0	0	0	0	NA	NA	NA	NA	NA	NA	NA
P. fraudulenta				J328Ps16	f/2−Si	1,200,000	10	3	0.00008	0.0008	0	0	0	0	0	0	0	NA	NA	NA	NA	NA	NA	NA
P. fraudulenta			CAWB128	Kiwa1	f/2	82,200,000	10	15	0.0005	0.0025	0	0	0	0	0	0	0	NA	NA	NA	NA	NA	NA	NA
P. galaxiae	clade B		CAWB153	PA014Ps01	f/2	121,500,000	10	5	0.0005	0.0025	0	0	0	0	0	0	0	NA	NA	NA	NA	NA	NA	NA
					f/2−Si	70,666,667	9	9	0.00005	0.0005	0	0	0	0	0	0	0	NA	NA	NA	NA	NA	NA	NA
					f/2−Si	4,560,000	43	10	0.00005	0.0005	0	0	0	0	0	0	0	NA	NA	NA	NA	NA	NA	NA
P. galaxiae	clade B		CAWB154	PA014Ps07	f/2	213,750,000	10	5	0.0005	0.0025	0	0	0	0	0	0	0	NA	NA	NA	NA	NA	NA	NA
					f/2−Si	39,733,333	9	9	0.00005	0.0005	0	0	0	0	0	0	0	NA	NA	NA	NA	NA	NA	NA
					f/2−Si	3,040,000	43	10	0.00005	0.0005	0	0	0	0	0	0	0	NA	NA	NA	NA	NA	NA	NA
P. hasleana	clade I		CAWB146	G007Ps01	f/2−Si	426,667	9	4	0.0002	0.002	0	0	0	0	0	0	0	NA	NA	NA	NA	NA	NA	NA
					f/2−Si	304,000	43	5	0.0003	0.003	0	0	0	0	0	0	0	NA	NA	NA	NA	NA	NA	NA
P. hasleana	clade I		CAWB147	G007Ps02	f/2−Si	1,866,667	9	4	0.00005	0.0005	0	0	0	0	0	0	0	NA	NA	NA	NA	NA	NA	NA
					f/2−Si	955,200	43	5	0.0001	0.001	0	0	0	0	0	0	0	NA	NA	NA	NA	NA	NA	NA
P. hasleana	clade II		CAWB156	J328Ps15	f/2−Si	8,026,667	10	3	0.00005	0.0005	0	0	0	0	0	0	0	NA	NA	NA	NA	NA	NA	NA
					f/2−Si	6,032,000	13	3	0.00005	0.0005	0	0	0	0	0	0	0	NA	NA	NA	NA	NA	NA	NA
					f/2−Si	6,336,000	43	4	0.00005	0.0005	0	0	0	0	0	0	0	NA	NA	NA	NA	NA	NA	NA
					K	23,386,667	12	3	0.00005	0.0005	0	0	0	0	0	0	0	NA	NA	NA	NA	NA	NA	NA
P. hasleana	clade II		CAWB155	J237Ps05	f/2−Si	11,792,000	10	3	0.00005	0.0005	0	0	0	0	0	0	0	NA	NA	NA	NA	NA	NA	NA
					f/2−Si	3,008,000	13	3	0.00005	0.0005	0	0	0	0	0	0	0	NA	NA	NA	NA	NA	NA	NA
					K	8,016,000	12	3	0.00005	0.0005	0	0	0	0	0	0	0	NA	NA	NA	NA	NA	NA	NA
P. multiseries			CAWB149	G001Ps03	f/2−Si	2,896,000	9	4	0.00005	0.0005	**1.65**	**0.012**	**0.045**	0	0	**0.020**	**0.003**	**95.4**	**0.7**	**2.6**	NA	NA	**1.2**	**0.2**
P. multiseries				G001Ps04	f/2−Si	3,024,000	10	8	0.00005	0.0005	**0.47**	**0.002**	**0.014**	**trace**	**0.012**	**0.003**	**trace**	**94.1**	**0.3**	**2.8**	**NC**	**2.3**	**0.5**	**NC**
P. multiseries				G007Ps04	f/2−Si	1,306,667	9	4	0.00008	0.0008	**2.05**	**NC[d]**	**0.059**	0	**NC[d]**	**0.012**	**0.002**	**96.5**	**NC**	**2.8**	NA	**NC**	**0.6**	**0.1**
P. multiseries				G010Ps10	f/2−Si	1,006,667	9	4	0.0001	0.001	**7.20**	0	**0.206**	0	**0.239**	**0.019**	**0.005**	**93.9**	NA	**2.7**	NA	**3.1**	**0.3**	**0.1**
P. multistriata		ST1	CAWB137	PA014Ps09	f/2	51,150,000	10	5	0.0005	0.0025	0	0	0	0	0	0	0	NA	NA	NA	NA	NA	NA	NA
P. multistriata		ST1		PA014Ps10	f/2	51,562,500	10	5	0.0005	0.0025	0	0	0	0	0	0	0	NA	NA	NA	NA	NA	NA	NA
					f/2−Si	10,506,667	8	11	0.00005	0.0005	0	0	0	0	0	0	0	NA	NA	NA	NA	NA	NA	NA
P. multistriata		ST1	CAWB150	PB024Ps01	f/2−Si	10,240,000	9	4	0.00005	0.0005	0	0	0	0	0	0	0	NA	NA	NA	NA	NA	NA	NA
P. multistriata		ST1		G013Ps21	f/2−Si	7,013,333	9	4	0.00005	0.0005	0	0	0	0	0	0	0	NA	NA	NA	NA	NA	NA	NA
P. multistriata		ST1		G027Ps06	f/2−Si	3,040,000	8	5	0.00005	0.0005	**trace**	0	**trace**	0	0	0	0	**NC**	NA	**NC**	NA	NA	NA	NA
P. multistriata		ST2	CAWB127	827	f/2	50,700,000	10	19	0.0005	0.0025	0	0	0	0	0	0	0	NA	NA	NA	NA	NA	NA	NA
P. multistriata		ST2		G001Ps01	f/2−Si	4,213,333	8	6	0.00005	0.0005	**trace**	0	**trace**	0	0	0	0	**NC**	NA	**NC**	NA	NA	NA	NA
P. multistriata		ST2		G001Ps02	f/2−Si	11,216,000	10	8	0.00005	0.0005	0	0	0	0	0	0	0	NA	NA	NA	NA	NA	NA	NA
P. plurisecta			CAWB151	G027Ps03	f/2−Si	7,733,333	9	3	0.00005	0.0005	0	0	0	0	0	0	0	NA	NA	NA	NA	NA	NA	NA
					f/2−Si	962,667	43	4	0.0001	0.001	0	0	0	0	0	0	0	NA	NA	NA	NA	NA	NA	NA
P. pungens	clade I	ST1	CAWB152	PA030Ps03	f/2−Si	953,333	9	4	0.0001	0.001	0	0	0	0	0	0	0	NA	NA	NA	NA	NA	NA	NA
P. pungens	clade I	ST1		G007Ps06	f/2−Si	3,536,000	10	8	0.00005	0.0005	0	0	0	0	0	0	0	NA	NA	NA	NA	NA	NA	NA
P. pungens	clade I	ST1		G005Ps01	f/2−Si	3,216,000	10	8	0.00005	0.0005	0	0	0	0	0	0	0	NA	NA	NA	NA	NA	NA	NA
P. pungens	clade I	ST1		G010Ps01	f/2−Si	3,744,000	10	8	0.00005	0.0005	0	0	0	0	0	0	0	NA	NA	NA	NA	NA	NA	NA
P. pungens	clade I	ST1		G018Ps05	f/2−Si	1,664,000	10	7	0.00006	0.0006	0	0	0	0	0	0	0	NA	NA	NA	NA	NA	NA	NA
P. pungens	clade I	ST1		Beatrix Bay	f/2	8,100,000	10	8	0.0005	0.0025	0	0	0	0	0	0	0	NA	NA	NA	NA	NA	NA	NA
P. pungens	clade I	ST1		G011Ps01	f/2−Si	1,368,000	9	4	0.00007	0.0007	0	0	0	0	0	0	0	NA	NA	NA	NA	NA	NA	NA
P. pungens	clade I	ST1		J328Ps21	f/2−Si	380,000	10	3	0.0003	0.003	0	0	0	0	0	0	0	NA	NA	NA	NA	NA	NA	NA
P. pungens	clade I	ST1		J237Ps03	f/2−Si	555,000	10	3	0.0002	0.002	0	0	0	0	0	0	0	NA	NA	NA	NA	NA	NA	NA
P. pungens	clade I	ST2		G027Ps05	f/2−Si	1,216,000	10	7	0.00008	0.0008	0	0	0	0	0	0	0	NA	NA	NA	NA	NA	NA	NA

P. pungens	clade I	ST2		G015Ps02	f/2−Si	1,824,000	10	7	0.00005	0.0005	0	0	0	0	0	0	0	NA	NA	NA	NA	NA	NA	NA
P. pungens	clade I	Unknown	CAWB129	Psd-3	f/2	16,020,000	10	13	0.0005	0.0025	0	0	0	0	0	0	0	NA	NA	NA	NA	NA	NA	NA
P. pungens	clade I	Unknown	CAWB130	Psd-4	f/2	8,700,000	10	13	0.0005	0.0025	0	0	0	0	0	0	0	NA	NA	NA	NA	NA	NA	NA
P. cf. *subpacifica*				PA030Ps02	f/2−Si	3,453,333	9	4	0.00005	0.0005	0	0	0	0	0	0	0	NA	NA	NA	NA	NA	NA	NA
P. cf. *subpacifica*				PA030Ps07	f/2−Si	3,440,000	10	8	0.00005	0.0005	0	0	0	0	0	0	0	NA	NA	NA	NA	NA	NA	NA
P. cf. *subpacifica*			CAWB148	PD009Ps04	f/2−Si	3,813,333	9	3	0.00005	0.0005	0	0	0	0	0	0	0	NA	NA	NA	NA	NA	NA	NA
P. cf. *subpacifica*				G011Ps03	f/2−Si	4,080,000	9	4	0.00005	0.0005	0	0	0	0	0	0	0	NA	NA	NA	NA	NA	NA	NA

[a] Sequences that were not identical but were not a clade/subclade level divergence were assigned as sequence type (ST).

[b] CICCM: the Cawthron Institute Culture Collection of Microalgae.

[c] The number of months took after isolation at the time of cultivation for DA and DA isomers analysis.

[d] Possible mix of epi-DA and iso-DA C was detected. These analogues co-eluted and could not be differentiated from each other for this sample.
